# RAB7L1-Mediated Relocalization of LRRK2 to the Golgi Complex Causes Centrosomal Deficits via RAB8A

**DOI:** 10.3389/fnmol.2018.00417

**Published:** 2018-11-13

**Authors:** Jesús Madero-Pérez, Belén Fernández, Antonio Jesús Lara Ordóñez, Elena Fdez, Evy Lobbestael, Veerle Baekelandt, Sabine Hilfiker

**Affiliations:** ^1^Institute of Parasitology and Biomedicine “López-Neyra”, Consejo Superior de Investigaciones Científicas, Granada, Spain; ^2^Laboratory for Neurobiology and Gene Therapy, KU Leuven, Leuven, Belgium

**Keywords:** LRRK2, RAB7L1, Golgi, centrosome, RAB8A, phosphorylation

## Abstract

Mutations in the LRRK2 gene cause autosomal-dominant Parkinson’s disease (PD), and both LRRK2 as well as RAB7L1 have been implicated in increased susceptibility to idiopathic PD. RAB7L1 has been shown to increase membrane-association and kinase activity of LRRK2, and both seem to be mechanistically implicated in the same pathway. Another RAB protein, RAB8A, has been identified as a prominent LRRK2 kinase substrate, and our recent work demonstrates that aberrant LRRK2-mediated phosphorylation of RAB8A leads to centrosomal alterations. Here, we show that RAB7L1 recruits LRRK2 to the Golgi complex, which causes accumulation of phosphorylated RAB8A in a pericentrosomal/centrosomal location as well as centrosomal deficits identical to those observed with pathogenic LRRK2. The centrosomal alterations induced by wildtype LRRK2 in the presence of RAB7L1 depend on Golgi integrity. This is in contrast to pathogenic LRRK2 mutants, which cause centrosomal deficits independent of Golgi integrity or largely independent on RAB7L1 expression. Furthermore, centrosomal alterations in the presence of wildtype LRRK2 and RAB7L1 are at least in part mediated by aberrant LRRK2-mediated RAB8A phosphorylation, as abolished by kinase inhibitors and reduced upon knockdown of RAB8A. These results indicate that pathogenic LRRK2, as well as increased levels of RAB7L1, cause centrosomal deficits in a manner dependent on aberrant RAB8A phosphorylation and centrosomal/pericentrosomal accumulation, suggesting that centrosomal cohesion deficits may comprise a useful cellular readout for a broader spectrum of the disease.

## Introduction

The LRRK2 gene encodes for a large cytosolic protein with kinase, GTPase and protein interaction domains. Mutations in LRRK2 cause familial autosomal-dominant forms of Parkinson’s disease (PD) (Paisan-Ruiz et al., 2004; Zimprich et al., 2004). In addition, genome-wide association studies have found common genetic variants at the LRRK2 locus that increase PD risk in non-familial, sporadic cases (Satake et al., 2009; Simon-Sanchez et al., 2009), suggesting that abnormal LRRK2 function is central to the entire PD disease spectrum (Cookson, 2017). Pathogenic mutations in LRRK2 cause increased kinase activity which can lead to cellular demise (West et al., 2005; Greggio et al., 2006; Smith et al., 2006; Lee et al., 2010; Steger et al., 2016), highlighting that the kinase activity of LRRK2 may represent an important therapeutic PD target.

LRRK2 is expressed in neuronal as well as non-neuronal cells and displays a broad subcellular distribution, suggesting that it performs role(s) shared by distinct cell types (Biskup et al., 2006). Indeed, LRRK2 has been reported to regulate endocytic, endolysosomal, autophagic and retromer-mediated intracellular trafficking events which all require polarized, microtubule-mediated transport (MacLeod et al., 2006, 2013; Soldati and Schliwa, 2006; Alegre-Abarrategui et al., 2009; Gomez-Suaga et al., 2012; Matta et al., 2012; Sanchez-Danes et al., 2012; Beilina et al., 2014; Gomez-Suaga et al., 2014). Alterations in polarized membrane trafficking may also underlie the consistently reported deficits in neurite outgrowth/cell polarity observed in pathogenic LRRK2-expressing cells (MacLeod et al., 2006, 2013; Dachsel et al., 2010; Winner et al., 2011; Sepulveda et al., 2013; Madero-Perez et al., 2018).

The centrosome is the primary microtubule-organizing center and plays a crucial role in cell polarity. It is tightly associated with the Golgi apparatus (Sutterlin and Colanzi, 2010; Rios, 2014), and the proper positioning and physical proximity between these two organelles is thought to contribute to endocytic and exocytic vesicular transport and polarized cargo delivery (Elric and Etienne-Manneville, 2014). Centrosomes are also important during the cell cycle by allowing the formation of a bipolar spindle required for chromosome segregation, and centrosome duplication and separation are tightly regulated processes (Nigg and Stearns, 2011). Our previous studies indicate that pathogenic LRRK2 causes deficits in centrosome positioning and cell polarity as well as centrosomal cohesion deficits in dividing cells, which seem to be at least in part due to the abnormal pericentrosomal/centrosomal accumulation of phosphorylated RAB8A (Madero-Perez et al., 2018). In addition, centrosomal cohesion deficits are observed in two distinct peripheral cell types derived from G2019S-LRRK2 PD patients as compared to healthy controls, and these defects are reverted by different LRRK2 kinase inhibitors, suggesting that the centrosomal cohesion phenotype may serve as a cellular biomarker in peripheral cells from PD patients to evaluate efficacy of LRRK2 kinase inhibitors in clinical settings (Madero-Perez et al., 2018).

RAB8A is a member of the RAB family of small GTPases which act as crucial regulators of distinct intracellular vesicular trafficking events (Hutagalung and Novick, 2011). RAB8A is localized to the Golgi and to a tubular early recycling compartment, and known to regulate post-Golgi exocytic membrane trafficking as well as endocytic recycling steps (Hattula et al., 2006; Peranen, 2011). RAB8A comprises one of the major LRRK2 kinase substrates identified to date (Steger et al., 2016), and its LRRK2-mediated phosphorylation has been suggested to impair ciliogenesis, another centrosome-related event requiring vesicular trafficking (Steger et al., 2017). Therefore, abnormal LRRK2-mediated RAB8A phosphorylation seems to be a key event underlying cellular alterations linked to abnormal centrosome functioning.

Human genetic studies have described common variants in loci other than LRRK2 which modify PD risk, such as the PARK16 locus (Satake et al., 2009; Simon-Sanchez et al., 2009; Lill et al., 2012). The PARK16 locus encompasses several genes including RAB7L1 (also called RAB29), another member of the RAB family. Transcriptome analysis has suggested that the PARK16 locus enhances RAB7L1 expression (Beilina et al., 2014). In addition, genetic variants at the LRRK2 and PARK16 loci seem to impact upon PD risk in a non-additive manner (MacLeod et al., 2013; Pihlstrom et al., 2015), suggesting a common mechanism of action. RAB7L1 has been implicated in *trans*-Golgi network integrity and in retromer-mediated trafficking between the Golgi and the endolysosomal compartment, which may affect lysosome integrity and axonal elongation (MacLeod et al., 2013; Beilina et al., 2014; Wang et al., 2014; Kuwahara et al., 2016). LRRK2 directly interacts with RAB7L1 (MacLeod et al., 2013; Beilina et al., 2014), and such interaction causes recruitment of LRRK2 to the Golgi complex, and concomitant kinase activation (Fujimoto et al., 2018; Liu et al., 2018; Purlyte et al., 2018). RAB7L1 has also been implicated in centrosome-related events (Onnis et al., 2015) known to be modulated by RAB8A (Nachury et al., 2007).

In the present study, we demonstrate that RAB7L1 recruits wildtype LRRK2 to the Golgi complex. Such RAB7L1-mediated recruitment is observed with wildtype, pathogenic mutant, as well as genetically or pharmacologically kinase-inhibited LRRK2, indicating that recruitment is independent of the LRRK2 kinase activity. The RAB7L1-mediated subcellular relocalization of wildtype LRRK2 is dependent on Golgi integrity and causes centrosomal cohesion deficits identical to those observed with pathogenic LRRK2, which are mediated by the LRRK2 kinase activity. The cohesion deficits depend, at least in part, on RAB8A, and correlate with the abnormal accumulation of phosphorylated RAB8A in a pericentrosomal/centrosomal location. Our findings suggest that RAB7L1 regulates LRRK2 Golgi localization with downstream effects on centrosomal behavior in a manner dependent on LRRK2-mediated RAB8A phosphorylation, and indicate that the centrosomal cohesion phenotype may comprise a cellular biomarker in peripheral patient-derived cells for a broader spectrum of PD.

## Materials and Methods

### DNA Constructs and Site-Directed Mutagenesis

GFP-tagged human LRRK2 constructs have been previously described (Madero-Perez et al., 2018). Myc-tagged LRRK1 and myc-tagged LRRK2 constructs have been previously described (Gomez-Suaga et al., 2014). DNA was prepared from bacterial cultures grown at 28°C (at 37°C for RAB constructs) using a midiprep kit (Promega) according to manufacturer’s instructions. mRFP-RAB7A has been previously described (Gomez-Suaga et al., 2014), and human dsRed-RAB9 was a gift from R. Pagano (Addgene plasmid #12677) (Choudhury et al., 2002). Human EGFP-RAB7L1 was kindly provided by Dr. J. Galán (Yale University School of Medicine, New Haven, CT, United States) (Spano et al., 2011). Human dsRed-RAB9 (containing two RAB9 sequences) was properly re-derived into pdsRed-Express-C1 (Clontech, #632430) by Gibson Assembly Master Mix (New England Biolabs). Human mRFP-RAB7L1 was generated by PCR amplification of the RAB7L1 coding sequence using EGFP-RAB7L1 as template, followed by subcloning into an mRFP vector backbone using XhoI-HindIII restriction enzyme sites. All triple-flag (3xFlag)-tagged RAB constructs, as well as GFP-tagged RAB7A and RAB9 constructs were generated using Gibson Assembly Master Mix (New England Biolabs). The RAB7A Q67L and T22N mutants, RAB9 Q66L and S21N mutants, RAB7L1 Q67L and T21N mutants and L728D/L729D-LRRK2 mutant were generated by site-directed mutagenesis (QuickChange, Stratagene), and the identity of all constructs was verified by sequencing of the entire coding region.

### Cell Culture and Transfections

HEK293T cells were cultured as described (Madero-Perez et al., 2018) and transfected at 80% confluence with 2 μg of LRRK2 constructs and 200 ng of RAB constructs where indicated, and 6 μl of LipoD293 (SignaGen Laboratories) per well of a 6-well plate for 5 h in full medium, resulting in a roughly 30% transfection/co-transfection efficiency, respectively. Cells were split 1:5 the following day, and processed for immunocytochemistry or Western blotting 48 h after transfection.

SH-SY5Y cells stably expressing GFP, flag-tagged wildtype LRRK2, or flag-tagged G2019S-mutant LRRK2 were cultured as described (Madero-Perez et al., 2018) and subcultured at a ratio of 1:6 twice a week. Transfection of cells was carried out at 80% confluence with 0.4 μg of DNA and 1.5 μl of Lipofectamine 2000 (Invitrogen) per well of a 24-well plate in 200 μl OptiMEM. Five hours later, cells were changed into full medium, passaged the following day at a 1:5 ratio onto coverslips, and fixed and stained 72 h after transfection.

Where indicated, cells were treated with brefeldin A (7.5 μg/ml, Sigma-Aldrich) or with nocodazole (200 nM, Sigma-Aldrich) for 3 h, or with 100 nM MLi2 (MRC PPU, Dundee, United Kingdom), 500 nM LRRK2-IN1 (obtained through the MJFF) or 500 nM GSK2578215A (Tocris) for 1 h before fixation.

### Immunofluorescence and Laser Confocal Imaging

Cells were fixed using 2% paraformaldehyde (PFA) in PBS for 20 min at room temperature, followed by permeabilization with 0.2% Triton-X100/PBS for 20 min. Coverslips were blocked for 1 h with 0.5% (w/v) BSA in 0.2% Triton-X100/PBS (blocking solution), followed by incubation with primary antibodies in blocking solution overnight at 4°C. Primary antibodies included rabbit polyclonal anti-pericentrin (Abcam, ab4448, 1:1000), mouse monoclonal anti-pericentrin (Abcam, ab28144, 1:1000), mouse monoclonal p230/Golgin-245 (Becton Dickinson, 611280, 1:400), rabbit polyclonal anti-RAB8A (Millipore, ABC423, 1:1000), knockout-validated rabbit monoclonal anti-RAB8A (Abcam, ab188574, 1:1000), rabbit polyclonal anti-T72-phospho-RAB8A (1:500, generous gift of D. Alessi, University of Dundee, United Kingdom), knockout-validated sheep polyclonal anti-RAB8A (S969D) and sheep polyclonal anti-T72-phospho-RAB8A (S874D) (MRC PPU). Sheep antibodies were used at a 1:50 dilution, and the anti-T72-phospho-RAB8A antibody was used in the presence of a 10-fold molar excess of dephospho-peptide, or of phospho-peptide where indicated. All double-immunocytochemistry involving sheep antibodies was performed sequentially, with the sheep antibodies employed first. Secondary antibodies included Alexa 405-conjugated goat anti-mouse or goat anti-rabbit, Alexa 488-conjugated goat anti-mouse or goat anti-rabbit, Alexa 594-conjugated goat anti-mouse or goat anti-rabbit, Alexa 647-conjugated goat anti-mouse or goat anti-rabbit (Invitrogen, 1:1000), Alexa 488-conjugated donkey anti-sheep (Invitrogen, 1:1000) or Alexa 594-conjugated donkey anti-sheep (Abcam, 1:1000). As indicated, cells were either mounted using mounting medium containing DAPI (Vector Laboratories), or incubated with TO-PRO-3 Iodide (642/661) (Invitrogen, 1:1000) for 3 min, followed by washes in PBS before mounting in ProLong Gold Antifade mounting medium (Invitrogen).

Images were acquired on a Leica TCS-SP5 confocal microscope using a 63× 1.4 NA oil UV objective (HCX PLAPO CS). Images were collected using single excitation for each wavelength separately and dependent on secondary antibodies (405 nm UV diode and a 415–455 nm emission band pass; 488 nm Argon Laser line and a 510–540 nm emission band pass; 543 HeNe laser line and a 600–630 nm emission band pass; 633 HeNe Laser line and a 640–670 nm emission band pass). GFP-tagged proteins were excited with 488 nm Argon Laser line and a 500–530 nm emission band pass, and RFP-tagged proteins with 543 nm HeNe Laser line and a 560–590 nm emission band pass, respectively. DAPI was excited with the 405 nm UV diode and a 430–480 nm emission band pass, and TO-PRO with 633 nm HeNe Laser line and a 650–675 nm emission band pass, respectively.

Ten to fifteen image sections of selected areas were acquired with a step size of 0.5 μm, and z-stack images analyzed and processed using Leica Applied Systems (LAS AF6000) image acquisition software. The same laser intensity settings and exposure times were used for image acquisition of individual experiments to be quantified. Centrosomes were scored as being separated when the distance between their centers was >1.5 μm (Madero-Perez et al., 2018), as analyzed by ImageJ software. 50 to 100 transfected cells per condition (exact numbers detailed in all figure legends) were analyzed in each experiment, and in all cases, mitotic cells were excluded from this analysis.

Total and phospho-RAB8A signals were scored as present or absent over non-processed and non-saturated images acquired during the same day with the same laser intensities, and around 100 transfected cells quantified per condition per experiment. Most experiments were quantified by an additional observer blind to conditions, with similar results obtained in all cases.

Re-localization of LRRK2 to the RAB7L1 compartment in HEK293T and SH-SY5Y cells was quantified visually as being recruited/not recruited, as no intermediate phenotypes could be observed. At least 50 transfected cells per condition were analyzed in at least three independent experiments.

The JACoP plugin of Fiji was used for the quantification of colocalization of the different GFP-tagged LRRK2 proteins with mRFP-tagged RAB7L1. The percentage of colocalization was obtained by calculating the Mander’s coefficient (M2) and expressed as M2 × 100. A total number of 10 independent cells were analyzed per condition per experiment.

### Knockdown of RAB8A or RAB7L1 by RNA Interference

Knockdowns were performed as previously described (Madero-Perez et al., 2018). Briefly, HEK293T cells were seeded in 6-well plates at 30–40% confluence 1 day prior to transfection such that they were at a confluence of 70–80% the following day. Cells were transfected with 40 nM siRNA (20 nM siRNA 1 + 20 nM siRNA 2) using 4 μl of jetPRIME Transfection Reagent (Polyplus-Transfection SA, no 114-15) in 200 μl jetPRIME buffer. The mix was incubated for 15 min at room temperature and added to 2 ml of full medium per well of a 6-well plate. Four hours later, media was replaced and cells transfected with 2 μg of the indicated LRRK2 constructs and 200 ng of the indicated RAB constructs and 6 μl of LipoD293 (SignaGen Laboratories) per well of a 6-well plate overnight in full medium. In all cases, cells were passaged 24 h later and processed for Western blot analysis or immunocytochemistry 48 h after transfection. RNAi reagents included Silencer Select Negative Control no. 1 siRNA (Ambion, Thermo Fisher, cat. nr 4390843; 50 nM) Silencer Select RAB8A (Ambion, Thermo Fisher, ID S8679), Silencer Select RAB8A (Ambion, Thermo Fisher, ID S8680), Silencer Select RAB7L1 (Ambion, Thermo Fisher, ID S17082) and Silencer Select RAB7L1 (Ambion, Thermo Fisher, ID S17083).

### Cell Extracts and Western Blotting

Cells were collected 48 h after transfection, washed in PBS and resuspended in cell lysis buffer (1% SDS in PBS containing 1 mM PMSF, 1 mM Na_3_VO_4_, 5 mM NaF). Extracts were sonicated, boiled, and centrifuged at 13,500 rpm for 10 min at 4°C. Protein concentration of supernatants was estimated using the BCA assay (Pierce), and 30 μg of extracts resolved by SDS-PAGE and analyzed by Western blot, using a rabbit polyclonal anti-GFP antibody (ab6556, 1:3000, Abcam), a knockout-validated sheep polyclonal anti-RAB8A (MRC PPU, S969D, 1:200), a sheep polyclonal anti-phospho-RAB8A (MRC PPU, S874D, 1:200), a knockout-validated rabbit monoclonal anti-RAB8A (Abcam, ab188574, 1:1000), a knockout-validated sheep polyclonal anti-RAB7L1 (MRC PPU, S984D, 1:250), a rabbit polyclonal anti-T72-phospho-RAB8A (1:500, generous gift of D. Alessi, University of Dundee, United Kingdom), a mouse monoclonal anti-flag antibody (clone M2, 1:2000, Sigma) and a mouse monoclonal anti-GAPDH (ab9484, 1:2000, Abcam) as loading control. Some Westerns were developed with ECL reagents (Roche), and a series of timed exposures to ensure that densitometric analyses were performed at exposures within the linear range. Quantification was performed using Quantity One software (Bio-Rad). The majority of Western blotting was performed according to the protocol described by LI-COR for Near-Infrared Western Blot Detection. In all cases, incubation with primary antibodies was performed overnight at 4°C, and secondary antibodies were incubated for 1 h at RT. For analysis of RAB8A or phospho-RAB8A levels using this technology, a knockout-validated rabbit monoclonal anti-RAB8A antibody (ab188574, Abcam, 1:1000), and a rabbit polyclonal phospho-RAB8A antibody (1:500, generously provided by D. Alessi, University of Dundee, United Kingdom) were employed. Blots were imaged using an Odyssey CLx system, and quantification was done using the instrument’s Image Studio software.

### Immunoprecipitation Assays

HEK293T cells were cultured as described and co-transfected at 80% confluence with 2 μg of LRRK2 constructs and 200 ng of RAB constructs as indicated, and 6 μl of LipoD293 (SignaGen Laboratories) per well of a 6-well plate for 5 h in full medium. The following day, cells were split into 100 mm tissue culture plates, and were collected 48 h after transfection. Cells were washed in PBS, followed by resuspension in 1 ml of lysis buffer [50 mM Tris-HCl, pH 7.6, 150 mM NaCl, 2 mM MgCl_2_, 1% Triton-X100, 1 mM DTT, protease inhibitor cocktail (Roche) and phosphatase inhibitor cocktail 3 (Sigma)], and incubated on a rotary wheel for 1 h at 4°C. Lysates were subsequently spun at 13,000 rpm for 10 min at 4°C, and protein concentration of supernatants estimated by BCA assay (Sigma), with 1 mg of total protein subjected to immunoprecipitation with a rabbit polyclonal anti-GFP antibody (Abcam, Ab6556, 1 μg per sample). Lysates were incubated with antibody for 2 h at 4°C, followed by addition of protein G Sepharose Fast Flow (Amersham) and incubation overnight at 4°C. The next day, beads were washed three times with lysis buffer, and bound proteins eluted with Laemmli sample buffer and heating at 95°C for 4 min prior to separation by SDS-PAGE and Western blotting as indicated above, using a mouse monoclonal anti-GFP antibody (Roche, 1:1000) or a mouse monoclonal anti-flag antibody (clone M2, 1:1000, Sigma), respectively.

### Statistical Analysis

All data are expressed as means ± SEM. Unless otherwise noted, data were analyzed by one-way ANOVA with Tukey’s *post hoc* test, and *p* < 0.05 was considered significant. Statistical details to all experiments can be found in the figure legends. ^∗∗∗∗^*p* < 0.001; ^∗∗∗^*p* < 0.005; ^∗∗^*p* < 0.01; ^∗^*p* < 0.05.

## Results

### RAB7L1 Recruits LRRK2 to the Golgi in a Manner Independent of the LRRK2 Kinase Activity

We first determined the localization of mRFP-tagged RAB7L1 constructs in HEK293T cells. RAB7L1 was localized to a perinuclear area largely overlapping with a *trans*-Golgi marker, whilst a mutant shown to lack GTP binding (RAB7L1-T21N) was more cytosolic (Appendix Figure A1A). Similarly, a mutant designed to impair GTP hydrolysis (RAB7L1-Q67L) but shown to display higher GTP/GDP dissociation rates was more cytosolic (Appendix Figure A1A), even though expressed to a lesser degree (Appendix Figure A1B). Thus, and as previously described (Beilina et al., 2014), both RAB7L1-Q67L and RAB7L1-S21N mutants seem to act as loss-of-function variants.

**FIGURE 1:**
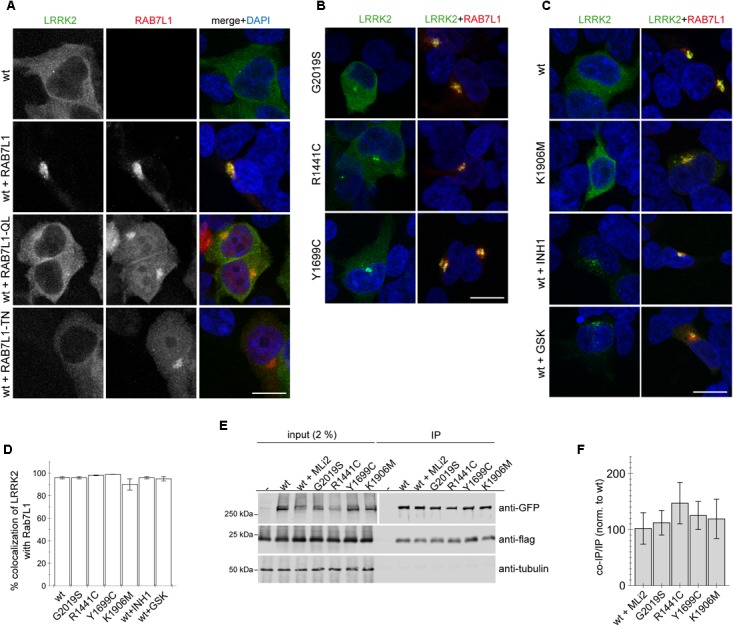
RAB7L1 causes recruitment of LRRK2 in a manner independent of kinase activity. **(A)** Example of HEK293T cells transfected with either GFP-tagged wildtype LRRK2 (green), or cotransfected with wildtype LRRK2 and wildtype or mutant mRFP-tagged RAB7L1 (red) as indicated, and stained with DAPI (blue). Scale bar, 10 μm. **(B)** Example of cells cotransfected with the indicated pathogenic GFP-LRRK2 mutants (green) and mRFP-RAB7L1 (red) as indicated, and stained with DAPI (blue). Scale bar, 10 μm. **(C)** Example of cells cotransfected with mRFP-RAB7L1 (red) and either wildtype or K1906M kinase-dead mutant GFP-LRRK2 (green), or cotransfected with wildtype GFP-LRRK2 (green) and treated with 500 nM LRRK2-IN-1 (INH1) or 500 nM GSK2578215A (GSK) for 60 min before fixation, and stained with DAPI (blue). Scale bar, 10 μm. **(D)** Quantification of the percentage of colocalization of the indicated LRRK2 constructs with RAB7L1 as determined by analysis of Mander’s coefficient (M2 × 100) from 10 random cells per condition. **(E)** Cells were cotransfected with the indicated GFP-tagged LRRK2 constructs and flag-tagged RAB7L1, and treated with 100 nM MLi2 for 60 min where indicated. Binding between LRRK2 and RAB7L1 was assessed by co-immunoprecipitation of GFP-tagged LRRK2 variants with flag-tagged RAB7L1 using a polyclonal GFP antibody. Left panel shows inputs, and right panel shows samples after immunoprecipitation, probed either for GFP (using a monoclonal GFP antibody), or in a separate gel probed for flag and tubulin as loading control. **(F)** Quantification of experiments as depicted in **(E)** were performed by comparing the amount of coimmunoprecipitated RAB7L1 to the amount of LRRK2 in the immunoprecipitation (IP). Data are mean ± SEM (*n* = 6 independent experiments).

When expressed on its own, wildtype GFP-tagged LRRK2 displayed a largely cytosolic localization (Figure 1A). In contrast, co-expression of wildtype LRRK2 with wildtype but not loss-of-function mutant RAB7L1 caused recruitment of LRRK2 to the RAB7L1 compartment in all co-transfected cells (Figure 1A). Such RAB7L1-mediated recruitment was observed with LRRK2, but not with LRRK1, which remained largely cytosolic also in the presence of RAB7L1 (Appendix Figure A2). Three distinct pathogenic LRRK2 mutants (G2019S, R1441C, Y1699C) were preferentially localized to the cytosol, but also to a pericentrosomal/centrosomal area (Figure 1B) (Madero-Perez et al., 2018). Coexpression with RAB7L1 caused the relocalization of all pathogenic LRRK2 mutants in all cells analyzed (Figure 1B). Similarly, cytosolic kinase-inactive K1906M mutant LRRK2, as well as pharmacologically kinase-inhibited LRRK2, which tends to localize to a pericentrosomal/centrosomal area as well as colocalize with microtubules (Dzamko et al., 2010; Deng et al., 2011; Kett et al., 2012; Blanca Ramirez et al., 2017; Madero-Perez et al., 2018), were recruited upon co-expression with RAB7L1 in all cells examined (Figure 1C). Co-localization analysis of LRRK2 with RAB7L1 indicated a high percentage of colocalization without differences between the distinct conditions (Figure 1D). When assessed by co-immunoprecipitation assays from detergent-solubilized extracts, 3xFlag-tagged RAB7L1 was found to similarly interact with wildtype, pathogenic mutant or kinase-inhibited LRRK2 (Figures 1E,F) (Beilina et al., 2014). In contrast, 3xFlag-tagged RAB7A or RAB9 did not interact with wildtype LRRK2 (Appendix Figures A3E,F). Thus, in a cellular context, RAB7L1 causes the relocalization of wildtype, pathogenic, kinase-dead as well as kinase-inhibited LRRK2, indicating that the kinase activity is not required for such RAB7L1-mediated recruitment of LRRK2.

### The RAB7L1-Mediated Recruitment of Wildtype LRRK2 Causes Centrosomal Cohesion Deficits

**FIGURE 2:**
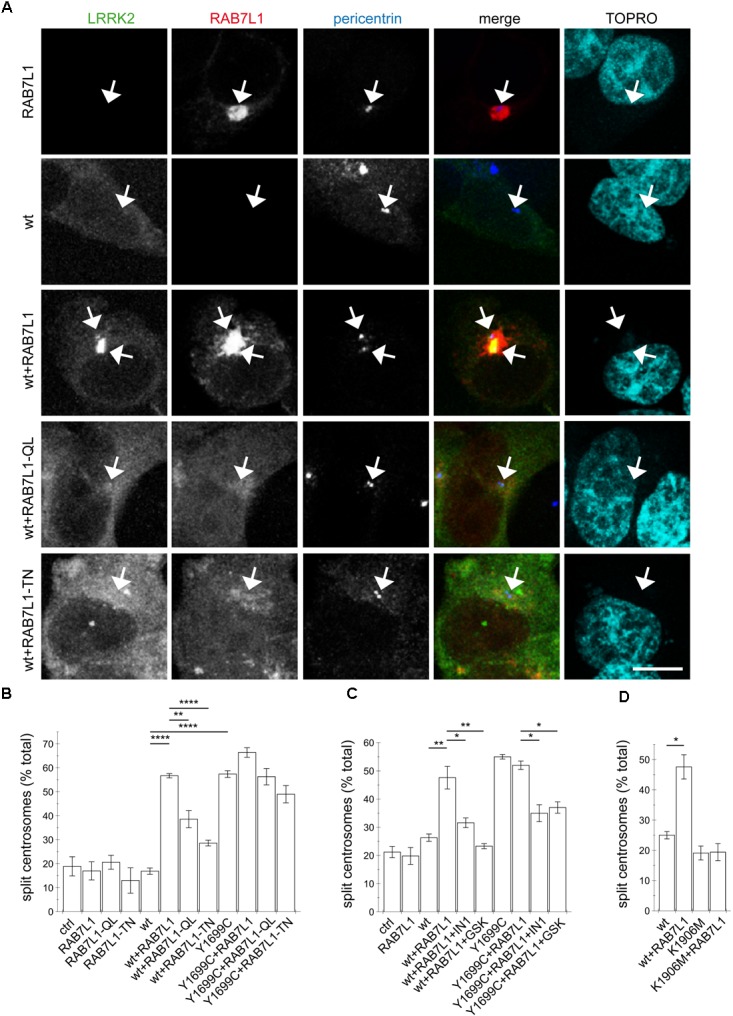
The RAB7L1-mediated recruitment of wildtype LRRK2 causes centrosomal cohesion deficits in a manner dependent on LRRK2 kinase activity and similar to those of pathogenic LRRK2. **(A)** Example of HEK293T cells transfected with either GFP-tagged wildtype LRRK2 (green), mRFP-tagged RAB7L1 (red), or a combination of wildtype LRRK2 and RAB7L1 or mutants thereof as indicated, and stained with pericentrin antibody (Alexa 405-conjugated secondary antibody, blue) and TO-PRO-3 (far red fluorescence similar to Alexa 647, pseudo-colored in cyan). Scale bar, 5 μm. **(B)** Quantification of the split centrosome phenotype in cells expressing RAB7L1 or mutants thereof, or co-expressing wildtype or Y1699C-mutant LRRK2 and RAB7L1 or mutants thereof, as indicated. At least 50 transfected cells were analyzed per condition per experiment. Bars represent mean ± SEM (*n* = 3 experiments); ^∗∗∗∗^*p* < 0.001; ^∗∗^*p* < 0.01. **(C)** Quantification of the split centrosome phenotype in cells expressing RAB7L1 or mutants thereof, or co-expressing wildtype or Y1699C-mutant LRRK2 and RAB7L1 or mutants thereof, and treated with LRRK2-IN-1 (500 nM) or GSK2578215A (500 nM) for 60 min as indicated. At least 50 transfected cells were analyzed per condition per experiment. Bars represent mean ± SEM (*n* = 3 experiments); ^∗∗^*p* < 0.01; ^∗^*p* < 0.05. **(D)** Quantification of the split centrosome phenotype in cells expressing wildtype or K1906M kinase-dead mutant LRRK2 and RAB7L1 as indicated. At least 50 transfected cells were analyzed per condition per experiment. Bars represent mean ± SEM (*n* = 3 experiments); ^∗^*p* < 0.05.

Our previous studies have revealed a centrosomal cohesion deficit in pathogenic LRRK2-expressing cells (Madero-Perez et al., 2018). We therefore wondered whether the RAB7L1-mediated recruitment of wildtype LRRK2 to the Golgi complex may also cause centrosomal alterations. In HEK293T cells, no centrosomal cohesion deficits were observed when active or inactive RAB7L1 mutants were expressed on their own (Figures 2A,B). However, when coexpressed with wildtype LRRK2, active but not inactive RAB7L1 mutants caused relocalization of LRRK2, concomitant with an increase in the percentage of split centrosomes (Figures 2A,B). No further cohesion deficits were observed when expressing active RAB7L1 with pathogenic LRRK2 (Figures 2B,C), possibly because the overexpression of this pathogenic LRRK2 mutant already caused a maximal centrosomal cohesion deficit in this cell type.

To determine whether the effects were specific to RAB7L1, we co-expressed LRRK2 with either RAB7A or RAB9, two RAB proteins involved in endolysosomal and/or retromer-mediated trafficking pathways (Huotari and Helenius, 2011; Guerra and Bucci, 2016; Kucera et al., 2016) and reported to be modulated and/or interact with LRRK2 (Dodson et al., 2012, 2014). Neither active nor inactive RAB7A nor RAB9 variants caused centrosomal cohesion deficits on their own, albeit expressed to comparable degrees (Appendix Figures A3A–C). RAB7A or RAB9 also did not cause centrosomal deficits when co-expressed with wildtype LRRK2, and did not alter the centrosomal deficits induced by pathogenic LRRK2 (Appendix Figures A3A,B). Expression of either RAB7A or RAB9 failed to cause recruitment of wildtype LRRK2 to the respective RAB7A/RAB9 compartments (Appendix Figure A3D). This was paralleled by a lack of detectable interaction between LRRK2 and RAB7A or RAB9, in contrast to the interaction observed with RAB7L1 (Appendix Figures A3E,F). Thus, and at least amongst the RAB proteins analyzed here, the subcellular relocalization of wildtype LRRK2 seems rather specific to RAB7L1 and is associated with centrosomal cohesion deficits identical to those previously described for all pathogenic LRRK2 mutants (Madero-Perez et al., 2018).

### The RAB7L1-Induced Centrosomal Cohesion Deficits of Wildtype LRRK2 Are Kinase Activity-Mediated

We next determined whether the RAB7L1-induced centrosomal deficits in the presence of wildtype LRRK2 are due to the kinase activity of LRRK2. Short-term application of two specific and structurally distinct LRRK2 kinase inhibitors (Deng et al., 2011; Reith et al., 2012) significantly reverted the centrosomal cohesion deficits induced in the presence of RAB7L1 and wildtype LRRK2, and a similar reversal was observed when co-expressing RAB7L1 with pathogenic LRRK2 (Figure 2C). Similarly, even though both are recruited to the RAB7L1 compartment (Figure 1), centrosomal cohesion deficits were induced when coexpressing RAB7L1 with wildtype LRRK2, but not with kinase-inactive K1906M mutant LRRK2, respectively (Figure 2D).

**FIGURE 3:**
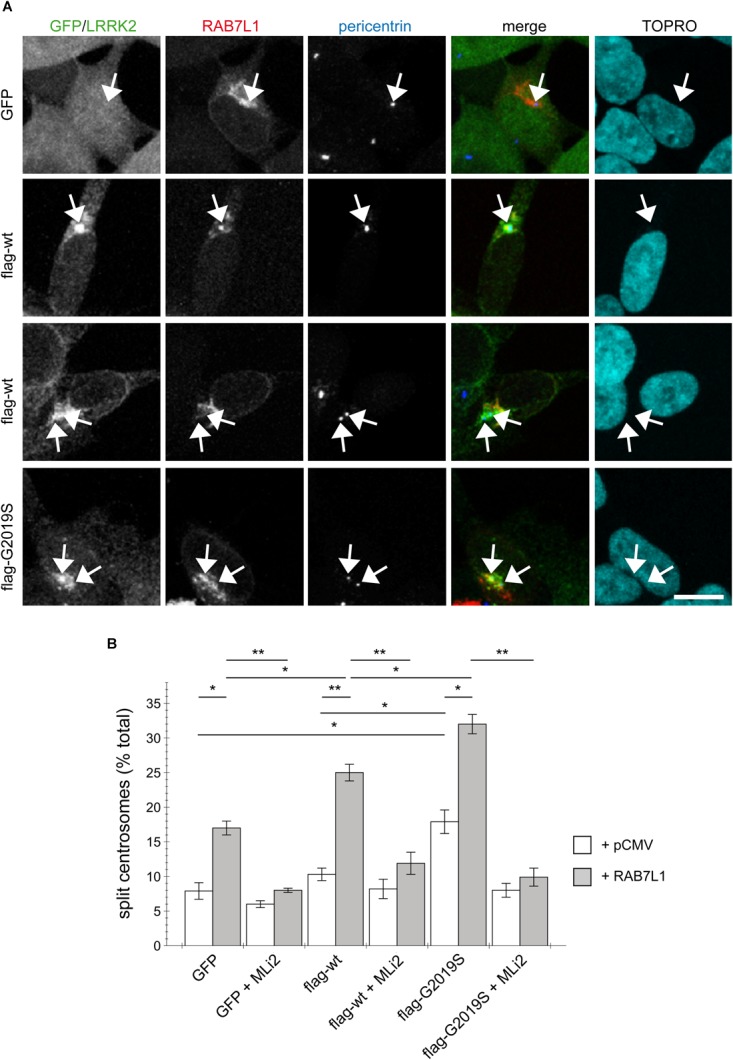
RAB7L1 potentiates the LRRK2-mediated centrosomal cohesion deficits in SH-SY5Y cells. **(A)** Example of SH-SY5Y cells stably expressing GFP (green), or flag-tagged wildtype or G2019S-mutant LRRK2 (Alexa 488-conjugated secondary antibody, green), and transiently transfected with mRFP-RAB7L1 (red), followed by staining for pericentrin (Alexa 405-conjugated secondary antibody, blue) and TO-PRO-3 (far red fluorescence similar to Alexa 647, pseudo-colored in cyan). Depicted are the most prominent centrosomal phenotypes, namely one centrosome in GFP-expressing cells, one centrosome or two split centrosomes in wildtype LRRK2-expressing cells, and two split centrosomes in G2019S mutant LRRK2-expressing cells, respectively. Scale bar, 10 μm. **(B)** Quantification of the split centrosome phenotype in SH-SY5Y cells expressing GFP, flag-tagged wildtype or G2019S-mutant LRRK2, and transfected with either empty vector (pCMV) or RAB7L1, and treated with MLi2 (100 nM) for 60 min as indicated. At least 100 transfected cells were analyzed per condition per experiment. Bars represent mean ± SEM (*n* = 3 experiments); ^∗∗^*p* < 0.01; ^∗^*p* < 0.05.

To probe for possible cell type-specific differences, we examined the effects of RAB7L1 expression in human SH-SY5Y neuroblastoma cells stably transduced with GFP, or with flag-tagged wildtype or G2019S mutant LRRK2, respectively (Reyniers et al., 2014; Vancraenenbroeck et al., 2014). As previously described (Madero-Perez et al., 2018), there was no difference in centrosome cohesion between control GFP and flag-tagged wildtype LRRK2-expressing cells, whilst the flag-tagged G2019S LRRK2-expressing cells displayed a bigger percentage of cells with split centrosomes (Figures 3A,B). In this cell system, expression of RAB7L1 on its own, in control cells and thus in the context of endogenous LRRK2 levels, caused a centrosomal cohesion phenotype (Figure 3B). Such RAB7L1-mediated enhancement of the centrosomal deficits was more pronounced in wildtype LRRK2-expressing cells as compared to control cells, and further enhanced in cells stably expressing G2019S mutant as compared to wildtype LRRK2, respectively (Figure 3B). In both wildtype and G2019S mutant LRRK2-expressing cells, RAB7L1 expression caused recruitment of the majority of LRRK2 to the RAB7L1 compartment (91 ± 4.5% recruitment in wildtype, 80 ± 4.7% recruitment in G2019S; *n* = 3 independent experiments), suggesting that the observed differences in centrosomal cohesion in the presence of Rab7L1 are due to the enhanced kinase activity of G2019S mutant versus wildtype LRRK2. Indeed, and in all cases examined, the centrosomal alterations were reverted upon short-term application of MLi2, a recently developed novel and highly selective LRRK2 kinase inhibitor (Fell et al., 2015), revealing that they were mediated by the LRRK2 kinase activity (Figure 3B). Therefore, expression of RAB7L1 in both HEK293T and SH-SY5Y cells causes the relocalization of wildtype and pathogenic LRRK2 to a RAB7L1 compartment largely overlapping with the *trans*-Golgi, and triggers centrosomal cohesion deficits which are dependent on the LRRK2 kinase activity.

### The Cohesion Deficits Induced by RAB7L1-Mediated LRRK2 Recruitment Correlate With Aberrant Centrosomal Accumulation of Phosphorylated RAB8A

**FIGURE 4:**
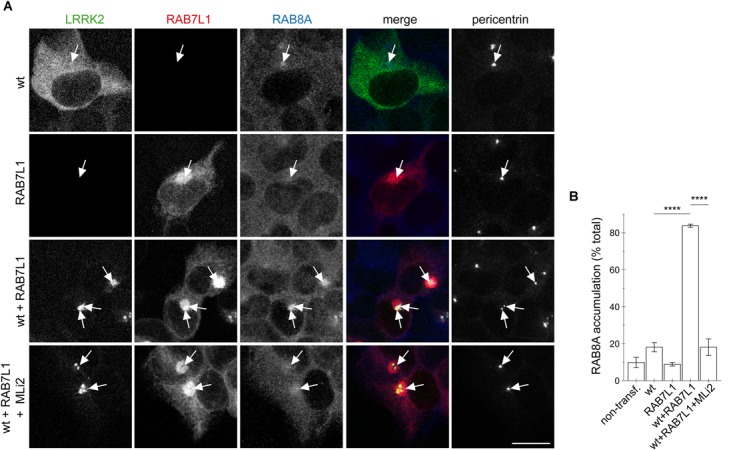
RAB7L1 causes kinase-dependent pericentrosomal/centrosomal accumulation of endogenous RAB8A in the presence of wildtype LRRK2. **(A)** Example of HEK293T cells transfected with either GFP-tagged wildtype LRRK2 (wt) (green), mRFP-tagged RAB7L1 (red), or a combination thereof, treated with 100 nM MLi2 for 60 min where indicated, and stained with RAB8A (Alexa 647-conjugated secondary antibody, pseudo-colored in blue) and with pericentrin antibody (Alexa 405-conjugated secondary antibody, pseudo-colored in gray). Scale bar, 10 μm. **(B)** Quantification of the percentage of transfected cells displaying pericentrosomal/centrosomal RAB8A staining of the type of experiments depicted in **(A)**. At least 50 transfected cells were analyzed per condition per experiment. Bars represent mean ± SEM (*n* = 3 experiments); ^∗∗∗∗^*p* < 0.001.

**FIGURE 5:**
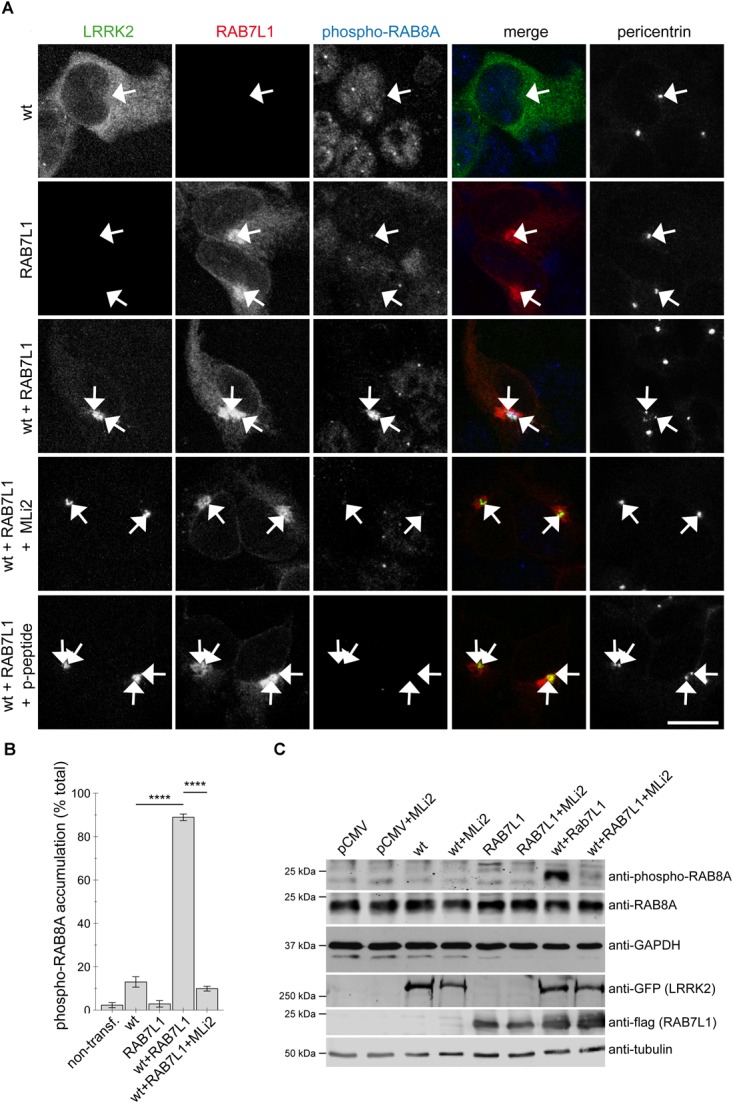
Coexpression of RAB7L1 and wildtype LRRK2 causes kinase-dependent pericentrosomal/centrosomal accumulation of endogenous phospho-RAB8A. **(A)** Example of HEK293T cells transfected with GFP-tagged wildtype LRRK2 (wt) (green), mRFP-tagged RAB7L1 (red), or a combination thereof, and stained with pericentrin (Alexa 405-conjugated secondary antibody, pseudo-colored in gray), and an anti-phospho-T72-RAB8A antibody (Alexa 647-conjugated secondary antibody, pseudo-colored in blue), the antibody preabsorbed with phospho-peptide (p-peptide), or cells treated with 100 nM MLi2 for 60 min prior to immunocytochemistry, as indicated. Scale bar, 10 μm. **(B)** Quantification of the percentage of non-transfected or transfected cells displaying phospho-RAB8A staining from experiments of the type depicted in **(A)**. At least 50 cells were analyzed per condition per experiment. Bars represent mean ± SEM (*n* = 3 experiments); ^∗∗∗∗^*p* < 0.001. **(C)** HEK293T cells were transfected with the different constructs and treated with 100 nM MLi2 for 60 min where indicated, and extracts blotted for phospho-RAB8A, total RAB8A, GFP (for LRRK2 detection), flag (for RAB7L1 detection), and GAPDH or tubulin as loading controls.

We previously showed that the centrosomal cohesion deficits induced by pathogenic LRRK2 are mediated by the aberrant pericentrosomal/centrosomal accumulation of phosphorylated RAB8A (Madero-Perez et al., 2018). We therefore next wondered whether the RAB7L1-mediated centrosomal alterations in the presence of wildtype LRRK2 may also be caused by the same mechanism. Co-expression of RAB7L1 with wildtype LRRK2 in HEK293T cells caused a prominent increase in the amount of cells displaying accumulation of pericentrosomal/centrosomal RAB8A as detected by a knockout-validated polyclonal sheep anti-RAB8A antibody (Steger et al., 2016) (Figures 4A,B). Such accumulation was reverted upon application of the LRRK2 kinase inhibitor MLi2 (Figures 4A,B), indicating that it may correspond to the phosphorylated form of RAB8A. When staining transfected cells with a polyclonal sheep antibody raised for the detection of phospho-T72-RAB8A (Steger et al., 2016), cells co-expressing RAB7L1 and wildtype LRRK2 displayed prominent centrosomal/pericentrosomal accumulation of phospho-RAB8A which was reversed when treating cells with MLi2, and which was not observed when preincubating the phospho-antibody with phospho-peptide (Figures 5A,B). Similarly, co-expression of RAB7L1 and wildtype LRRK2 caused detectable phosphorylation of endogenous RAB8A as measured by Western blotting, without changes in total RAB8A protein levels (Figure 5C). Such phospho-RAB8A signal was abolished when treating cells with kinase inhibitor, and was not observed when expressing wildtype LRRK2 alone (Figure 5C). Whilst accumulation of phospho-RAB8A by immunocytochemistry in SH-SY5Y cells stably expressing pathogenic LRRK2 could only be detected when overexpressing RAB8A (Madero-Perez et al., 2018), expression of RAB7L1 caused a detectable accumulation of endogenous phospho-RAB8A in SH-SY5Y cells expressing wildtype LRRK2, and a further accumulation in cells expressing G2019S-mutant LRRK2 (20.7 ± 5% of wildtype versus 50 ± 3.7% of G2019S-mutant LRRK2 cells; *n* = 3 experiments, *p* < 0.05; Appendix Figure A4). Thus, increasing the levels of RAB7L1 causes accumulation of endogenous phospho-RAB8A and deficits in centrosomal functioning in the presence of wildtype as well as pathogenic LRRK2.

**FIGURE 6:**
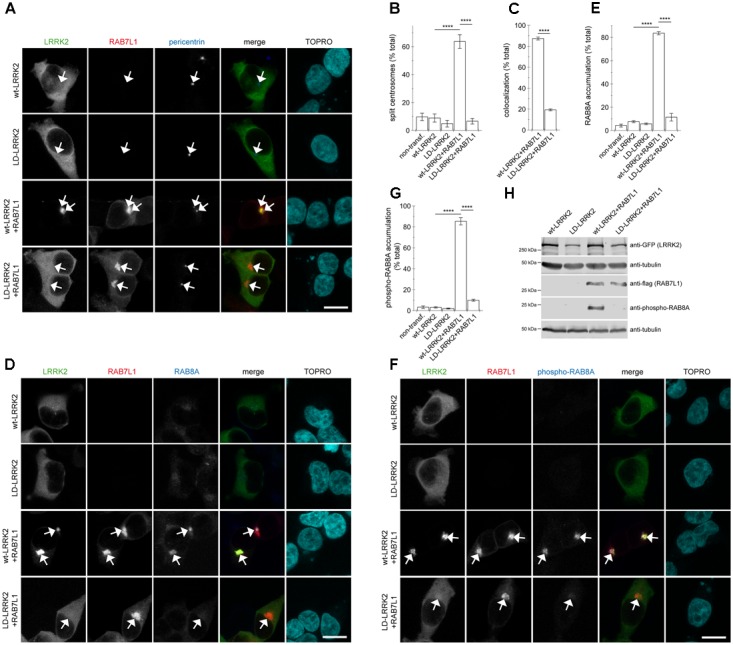
Coexpression of RAB7L1 with ankyrin domain residue mutant LRRK2 does not cause centrosomal deficits or pericentrosomal/centrosomal accumulation of endogenous phospho-RAB8A. **(A)** Example of HEK293T cells transfected with either GFP-tagged wildtype LRRK2 or LD-LRRK2 (green) in the presence or absence of mRFP-tagged RAB7L1 (red) as indicated, and stained with pericentrin antibody (Alexa 405-conjugated secondary antibody, blue) and TO-PRO-3 (far red fluorescence similar to Alexa 647, pseudo-colored in cyan). Scale bar, 10 μm. **(B)** Quantification of the split centrosome phenotype in non-transfected cells or cells expressing wildtype LRRK2 or LD-LRRK2 in the presence or absence of RAB7L1 as indicated. At least 50 transfected cells were analyzed per condition per experiment. Bars represent mean ± SEM (*n* = 3 experiments); ^∗∗∗∗^*p* < 0.001. **(C)** Quantification of recruitment of either wildtype LRRK2 or LD-LRRK2 to mRFP-RAB7L1 compartment. Around 100 transfected cells were analyzed per condition per experiment. Bars represent mean ± SEM (*n* = 3 experiments); ^∗∗∗∗^*p* < 0.001. **(D)** Example of HEK293T cells transfected with constructs as indicated, and stained with RAB8A antibody (Alexa 405-conjugated secondary antibody, blue) and TO-PRO-3 (far red fluorescence similar to Alexa 647, pseudo-colored in cyan). Scale bar, 10 μm. **(E)** Quantification of the percentage of non-transfected or transfected cells displaying pericentrosomal/centrosomal RAB8A staining of the type of experiments depicted in **(D)**. At least 50 transfected cells were analyzed per condition per experiment. Bars represent mean ± SEM. (*n* = 3 experiments); ^∗∗∗∗^*p* < 0.001. **(F)** Example of HEK293T cells transfected with constructs as indicated, and stained with an anti-phospho-T72-RAB8A antibody (Alexa 405-conjugated secondary antibody, blue) and TO-PRO-3 (far red fluorescence similar to Alexa 647, pseudo-colored in cyan). Scale bar, 10 μm. **(G)** Quantification of the percentage of non-transfected or transfected cells displaying phospho-RAB8A staining from experiments of the type depicted in **(F)**. At least 50 cells were analyzed per condition per experiment. Bars represent mean ± SEM (*n* = 3 experiments); ^∗∗∗∗^*p* < 0.001. **(H)** HEK293T cells were transfected with the indicated constructs, and extracts blotted for phospho-RAB8A, GFP (for LRRK2 detection), flag (for RAB7L1 detection), and tubulin as loading control.

To obtain additional evidence that the observed centrosomal defects were due to a RAB7L1-mediated recruitment of LRRK2 to the Golgi complex, we generated a double-point mutant (L728D/L729D) version in the ankyrin domain of LRRK2 (LD-LRRK2) previously reported to impair the RAB7L1-mediated membrane recruitment and activation of LRRK2 (Purlyte et al., 2018). When coexpressed with RAB7L1, wildtype LRRK2 was recruited to a RAB7L1 compartment which was associated with a pronounced centrosomal cohesion deficit, whilst LD-LRRK2 remained largely cytosolic and was without effect on centrosomal cohesion (Figures 6A–C). Similarly, expression of RAB7L1 with wildtype LRRK2 caused a pronounced accumulation of pericentrosomal/centrosomal total RAB8A as detected using a knockout-validated rabbit monoclonal RAB8A antibody, and accumulation of phospho-RAB8A as detected using a rabbit polyclonal anti-phospho-RAB8A antibody, respectively, which was not observed with the LD-LRRK2 mutant (Figures 6D–G). In addition, and as previously described (Purlyte et al., 2018), the presence of RAB7L1 in the context of wildtype, but not LD-mutant LRRK2, caused phosphorylation of RAB8A as determined by Western blotting techniques (Figure 6H). Therefore, it is the RAB7L1-mediated Golgi recruitment of LRRK2, rather than another RAB7L1-related function, which is responsible for the observed centrosomal cohesion deficits in the presence of wildtype LRRK2.

### The RAB7L1-Induced Centrosomal Deficits of Wildtype LRRK2 Require Golgi Integrity and Are RAB8A-Mediated

**FIGURE 7:**
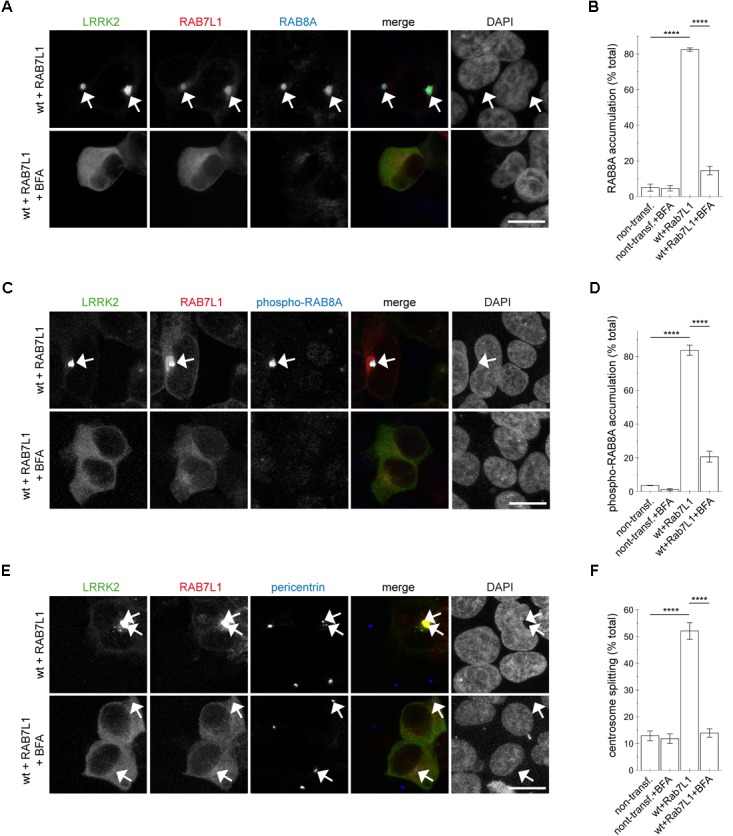
Integrity of the Golgi complex is required for the RAB7L1-mediated phospho-RAB8A accumulation and centrosomal cohesion deficits in the presence of wildtype LRRK2. **(A)** HEK293T cells were cotransfected with GFP-tagged LRRK2 (green) and mRFP-tagged RAB7L1 (red), and either treated or non-treated with brefeldin A (BFA; 7.5 μg/ml) for 3 h as indicated before staining with a knockout-validated rabbit monoclonal total RAB8A antibody (Alexa 647-conjugated secondary antibody, pseudo-colored in blue) and DAPI (pseudo-colored in gray). Scale bar, 10 μm. **(B)** Quantification of the percentage of non-transfected or transfected cells displaying pericentrosomal/centrosomal RAB8A staining, in the presence or absence of brefeldin A as indicated, from experiments of the type depicted in **(A)**. At least 50 cells were analyzed per condition per experiment. Bars represent mean ± SEM (*n* = 3 experiments); ^∗∗∗∗^*p* < 0.001; ^∗∗∗^ *p* < 0.005. **(C)** HEK293T cells were transfected as indicated, and either treated or non-treated with brefeldin A as described above, before staining with a rabbit polyclonal phospho-RAB8A antibody (Alexa 647-conjugated secondary antibody, pseudo-colored in blue) and DAPI (pseudo-colored in gray). Scale bar, 10 μm. **(D)** Quantification of the percentage of non-transfected or transfected cells displaying phospho-RAB8A staining, in the presence or absence of brefeldin A as indicated, from experiments of the type depicted in **(C)**. At least 50 cells were analyzed per condition per experiment. Bars represent mean ± SEM (*n* = 3 experiments); ^∗∗∗∗^*p* < 0.001. **(E)** Cells were transfected as indicated, and either treated or non-treated with brefeldin A as described above, before staining with pericentrin antibody (Alexa 647-conjugated secondary antibody, pseudo-colored in blue) and DAPI (pseudo-colored in gray). Scale bar, 10 μm. **(F)** Quantification of the split centrosome phenotype in non-transfected or transfected cells as indicated, in the presence or absence of brefeldin A, from experiments of the type depicted in **(E)**. At least 50 cells were analyzed per condition per experiment. Bars represent mean ± SEM (*n* = 3 experiments); ^∗∗∗∗^*p* < 0.001.

To determine whether Golgi integrity was required for the RAB7L1-mediated effects in the presence of wildtype LRRK2, cells were treated with brefeldin A, which causes redistribution of the Golgi complex into the ER (Lippincott-Schwartz et al., 1989; Copeland et al., 2016). Brefeldin A treatment caused loss of *trans*-Golgi staining in all non-transfected and the majority of mRFP-RAB7L1-expressing cells, respectively (Appendix Figures A5A–C). This correlated with the relocalization of RAB7L1 to the cytosol in the majority of cells, further confirming the *trans*-Golgi localization of expressed RAB7L1 (Appendix Figures A5A–C). In cells expressing RAB7L1 and wildtype LRRK2, brefeldin A treatment disrupted the perinuclear localization of RAB7L1 as well as LRRK2, which was associated with the loss of total RAB8A accumulation (Figures 7A,B). Similarly, brefeldin A treatment caused a pronounced loss of phospho-RAB8A accumulation in cells expressing RAB7L1 and wildtype LRRK2 (Figures 7C,D), which was paralleled by the reversal of the centrosomal cohesion deficits induced by RAB7L1 and wildtype LRRK2 (Figures 7E,F). Therefore, Golgi integrity is required for the centrosomal cohesion deficits mediated by RAB7L1 and wildtype LRRK2.

**FIGURE 8:**
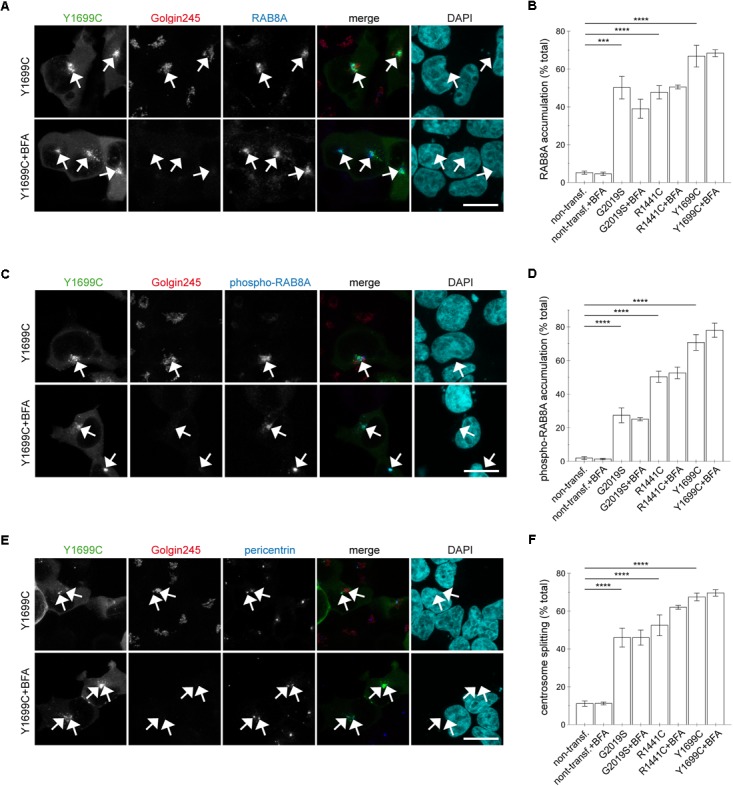
Integrity of the Golgi complex is not required for the pathogenic LRRK2-mediated phospho-RAB8A accumulation and centrosomal cohesion deficits. **(A)** HEK293T cells were transfected with Y1699C-mutant LRRK2 (green), either treated or non-treated with brefeldin A (BFA; 7.5 μg/ml) for 3 h, and stained with a knockout-validated rabbit monoclonal anti-RAB8A antibody (Alexa 647-conjugated secondary antibody, pseudo-colored in blue), a *trans*-Golgi marker antibody (Golgin245, Alexa 594-conjugated secondary antibody, red) and DAPI (pseudo-colored in cyan). Scale bar, 10 μm. **(B)** Quantification of the percentage of non-transfected cells, or cells transfected with the various pathogenic LRRK2 constructs displaying pericentrosomal/centrosomal RAB8A staining in the presence or absence of brefeldin A as indicated, from experiments of the type depicted in **(A)**. At least 50 cells were analyzed per condition per experiment. Bars represent mean ± SEM (*n* = 3 experiments); ^∗∗∗∗^*p* < 0.001; ^∗∗∗^*p* < 0.005. **(C)** Cells were transfected with Y1699C-mutant LRRK2 (green), and either treated or non-treated with brefeldin A as described above before staining with a rabbit polyclonal phospho-RAB8A antibody (Alexa 647-conjugated secondary antibody, pseudo-colored in blue), a *trans*-Golgi marker antibody (Golgin245, Alexa 594-conjugated secondary antibody, red) and DAPI (pseudo-colored in cyan). Scale bar, 10 μm. **(D)** Quantification of the percentage of non-transfected or transfected cells displaying phospho-RAB8A staining, in the presence or absence of brefeldin A as indicated, from experiments of the type depicted in **(C)**. At least 50 cells were analyzed per condition per experiment. Bars represent mean ± SEM (*n* = 3 experiments); ^∗∗∗∗^*p* < 0.001. **(E)** Cells were transfected with Y1699C-mutant LRRK2 (green), and either treated or non-treated with brefeldin A as described above before staining with a pericentrin antibody (Alexa 647-conjugated secondary antibody, pseudo-colored in blue), a *trans*-Golgi marker antibody (Golgin245, Alexa 594-conjugated secondary antibody, red) and DAPI (pseudo-colored in cyan). Scale bar, 10 μm. **(F)** Quantification of the split centrosome phenotype in non-transfected or transfected cells as indicated, in the presence or absence of brefeldin A, from experiments of the type depicted in **(E)**. At least 50 cells were analyzed per condition per experiment. Bars represent mean ± SEM (*n* = 3 experiments); ^∗∗∗∗^*p* < 0.001.

**FIGURE 9:**
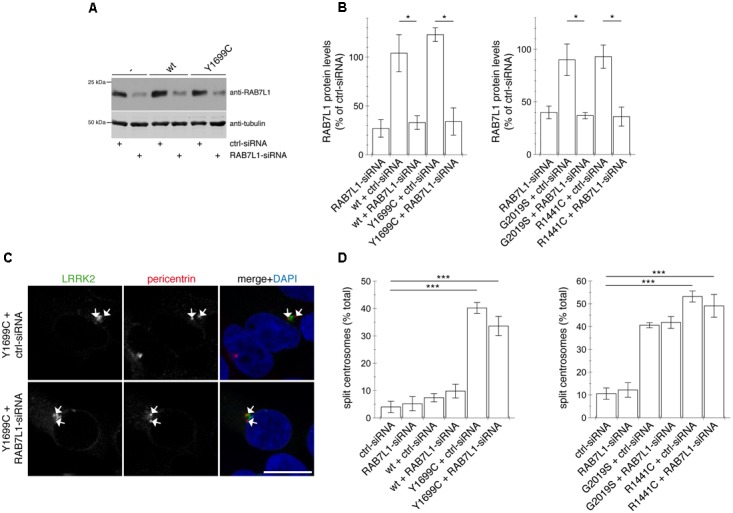
Knockdown of RAB7L1 does not alter the centrosomal cohesion deficits mediated by pathogenic LRRK2. **(A)** Representative Western blot of extracts from control cells (-), or cells transfected with wildtype (wt) or Y1699C-mutant LRRK2, along with either ctrl-siRNA or RAB7L1-siRNA as indicated, and blotted against RAB7L1 using a knockout-validated antibody, or tubulin as loading control. **(B)** Quantification of the type of experiments depicted in **(A)**, with levels of RAB7L1 normalized to tubulin and to RAB7L1 levels in the presence of ctrl-siRNA. Bars represent ± SEM (*n* = 3 experiments); ^∗^*p* < 0.05. **(C)** Example of HEK293T cells co-transfected with GFP-tagged Y1699C mutant LRRK2 (green) and either ctrl-siRNA or RAB7L1-siRNA as indicated, and stained with pericentrin antibody (Alexa 647-conjugated secondary antibody, pseudo-colored in red) and DAPI (blue). Scale bar, 10 mm. **(D)** Quantification of the split centrosome phenotype in control cells transfected with either ctrl-siRNA or RAB7L1-siRNA, or in cells co-transfected with siRNA and with wildtype or mutant LRRK2. Around 100 transfected cells were analyzed per condition per experiment. Bars represent mean ± SEM (*n* = 3 experiments); ^∗∗∗^*p* < 0.005.

This was in contrast to the effects induced by the three pathogenic LRRK2 mutants (G2019S, R1441C, Y1699C). Brefeldin A treatment caused a loss of *trans*-Golgi staining, but did not alter the accumulation of RAB8A (Figures 8A,B and Appendix Figures A6A, A7A) or phospho-RAB8A (Figures 8C,D and Appendix Figures A6B, A7B), and neither reverted the centrosomal cohesion deficits induced by any of the three pathogenic mutants (Figures 8E,F and Appendix Figures A6C, A7C). In agreement with these findings, RNAi of RAB7L1 with two different small interfering RNAs (siRNAs) did not alter the localization nor the centrosomal cohesion deficits mediated by the three pathogenic LRRK2 mutants (Figure 9 and Appendix Figure A8), suggesting that pathogenic LRRK2 causes centrosomal defects in a manner largely independent of RAB7L1 or of Golgi integrity. These data suggest that pathogenic LRRK2 may phosphorylate a pool of RAB8A distinct from the Golgi-resident RAB8A pool targeted by RAB7L1 and wildtype LRRK2. Indeed, brefeldin A treatment caused the cytosolic relocalization of mRFP-RAB8A in a much smaller percentage of cells as compared to mRFP-RAB7L1, consistent with previous reports that RAB8A localizes to the *trans*-Golgi, but also to an endocytic recycling compartment (Appendix Figures A5D–F).

**FIGURE 10:**
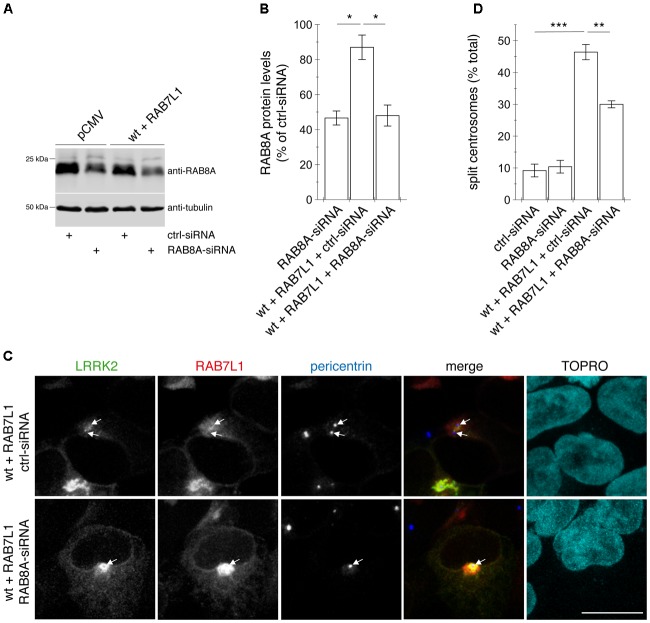
Knockdown of RAB8A significantly reverses the centrosomal cohesion deficits mediated by RAB7L1 and wildtype LRRK2. **(A)** Representative Western blot of extracts from control pCMV-transfected cells, or cells transfected with wildtype (wt) LRRK2 and RAB7L1, along with either ctrl-siRNA or RAB8A-siRNA as indicated, and blotted against RAB8A or tubulin as loading control. **(B)** Quantification of the type of experiments depicted in **(A)**, with levels of RAB8A normalized to tubulin and to RAB8A levels in the presence of ctrl-siRNA. Bars represent ± SEM (*n* = 3 experiments); ^∗^*p* < 0.05. **(C)** Example of HEK293T cells co-transfected with GFP-tagged wildtype LRRK2 (green) and mRFP-tagged RAB7L1 (red), and either with ctrl-siRNA or RAB8A-siRNA as indicated, and stained with pericentrin antibody (Alexa 405-conjugated secondary antibody, blue) and TO-PRO-3 (far red fluorescence similar to Alexa 647, pseudo-colored in cyan). Scale bar, 10 μm. **(D)** Quantification of the split centrosome phenotype in control cells transfected with either ctrl-siRNA or RAB8A-siRNA, or in cells co-transfected with the respective siRNAs and with wildtype LRRK2 and RAB7L1. Around 100 transfected cells were analyzed per condition per experiment. Bars represent ± SEM (*n* = 3 experiments); ^∗∗∗^*p* < 0.005; ^∗∗^*p* < 0.01.

Finally, to determine whether the centrosomal cohesion deficits caused by RAB7L1 and wildtype LRRK2 were RAB8A-dependent, HEK293T cells were transiently transfected with siRNA directed against a control sequence or with two different siRNAs against RAB8A, and knockdown of protein levels confirmed by Western blotting (Figures 10A,B). Knocking down RAB8A did not cause alterations in centrosomal cohesion in non-transfected cells, but caused a significant reversal of the cohesion deficits induced by the co-expression of RAB7L1 and LRRK2, without interfering with the recruitment of LRRK2 to the Golgi complex (Figures 10C,D). Therefore, and as described for pathogenic LRRK2 mutants (Madero-Perez et al., 2018), phosphorylated RAB8A seems to mediate, at least in part, the centrosomal cohesion deficits induced by RAB7L1 and wildtype LRRK2.

## Discussion

We show here that increasing the levels of RAB7L1 causes recruitment of wildtype LRRK2 to the Golgi complex. Such recruitment is independent of LRRK2 kinase activity, as kinase-dead mutant LRRK2 or pharmacologically kinase-inhibited LRRK2 display a similar RAB7L1-mediated Golgi recruitment. The RAB7L1-mediated recruitment of wildtype LRRK2 causes centrosomal cohesion deficits similar to the ones caused by three distinct pathogenic LRRK2 mutants (Madero-Perez et al., 2018). These defects are mediated by the LRRK2 kinase activity, as reverted upon pharmacological kinase inhibition and not observed with kinase-dead mutant LRRK2. In addition, the centrosomal defects depend on the capacity of RAB7L1 to recruit LRRK2 to the Golgi complex, as not observed when expressing RAB7L1 together with a LRRK2 mutant unable to interact with RAB7L1. The centrosomal deficits further depend on Golgi integrity, as not observed when expressing RAB7L1 and wildtype LRRK2 in cells treated with brefeldin A. Thus, both the RAB7L1-mediated Golgi recruitment as well as the kinase activity of wildtype LRRK2 are required for the observed centrosomal defects. These data are in agreement with recent studies indicating that RAB7L1 not only acts to recruit LRRK2 to the Golgi complex, but also triggers its kinase activation (Liu et al., 2018; Purlyte et al., 2018), even though the downstream cellular effects of such recruitment and activation have remained unknown. Pathogenic R1441C and Y1699C LRRK2 have been shown to be more activated by RAB7L1 as compared to G2019S or wildtype LRRK2 (Purlyte et al., 2018). In contrast, our data suggest that all three pathogenic mutants cause centrosomal cohesion deficits independent of Golgi integrity, and largely independent of RAB7L1. Thus, a RAB7L1-mediated activation of distinct pathogenic LRRK2 mutants at the Golgi complex seems not to be necessary, at least for this specific cellular readout.

We also analyzed the effects of RAB7L1 expression on centrosomal cohesion deficits in the context of endogenous levels of wildtype LRRK2. Whilst RAB7L1 expression was without effect in kidney-derived HEK293T cells, it caused a significant deficit in centrosomal cohesion when expressed in control SH-SY5Y cells. Whilst further work will be required to dissect the reasons for these cell type-specific differences, since increased PD risk seems to correlate with increased RAB7L1 expression (Beilina et al., 2014), our findings may have implications for targeting LRRK2 kinase activity also in RAB7L1-related idiopathic PD. Importantly, our data further suggest that centrosomal cohesion deficits may comprise a valid cellular biomarker readout for testing the efficacy of LRRK2 kinase inhibitors in clinical trials, as centrosomal cohesion deficits are also observed in distinct peripheral cell types derived from G2019S LRRK2-PD patients as compared to healthy controls (Madero-Perez et al., 2018). Since peripheral cells from patients with RAB7L1 PD risk factor variants are not currently available, we here employed cellular models with artificially increased levels of RAB7L1, which caused centrosomal deficits similar to those observed with pathogenic LRRK2.

**FIGURE 11:**
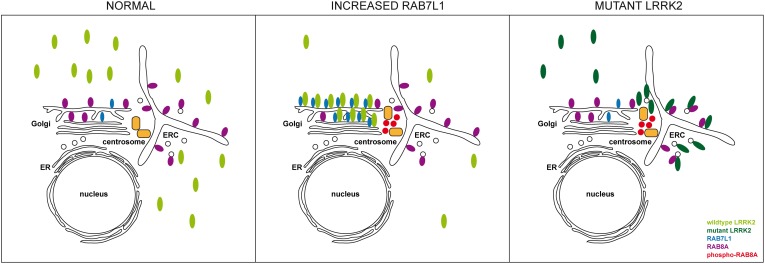
Model for how altered RAB7L1 levels and pathogenic LRRK2 cause centrosomal alterations via RAB8A phosphorylation. Under normal conditions, wildtype LRRK2 is largely cytosolic. Under conditions of increased RAB7L1 levels (increased RAB7L1), wildtype LRRK2 becomes recruited to the Golgi apparatus, where it phosphorylates Golgi-resident RAB8A. Phosphorylated RAB8A accumulates at/around the centrosome, possibly via binding to RILPL1/RILPL2, resulting in centrosomal cohesion deficits. Pathogenic LRRK2 (mutant LRRK2) displays a more pericentrosomal/centrosomal localization by currently unknown mechanisms, but possibly involving enhanced binding to RAB8A on the early recycling compartment and/or perinuclear vesicular structures. This causes RAB8A phosphorylation in those compartments, with phosphorylated RAB8A then accumulating at/around the centrosome to cause centrosomal cohesion deficits in a manner independent on Rab7L1 or on Golgi integrity. ER, endoplasmic reticulum; ERC, early recycling compartment.

The RAB7L1-mediated recruitment of wildtype LRRK2 to the Golgi complex likely causes the phosphorylation of a pool of Golgi-resident RAB8A, as phospho-RAB8A accumulation and centrosomal cohesion deficits were abolished upon disrupting Golgi complex integrity. In contrast, phospho-RAB8A accumulation and centrosomal cohesion deficits mediated by the three pathogenic LRRK2 mutants occurred independent of Golgi integrity or largely independently of RAB7L1, indicating that pathogenic LRRK2 causes the phosphorylation of another, non-Golgi-resident pool of RAB8A. RAB8A is also localized to a perinuclear early recycling compartment (Hattula et al., 2006; Peranen, 2011; Vaibhava et al., 2012). In addition, and in agreement with our data suggesting that pathogenic LRRK2 variants localize to a perinuclear area, recent studies have shown that membrane-bound pathogenic LRRK2 largely colocalizes with RAB8A in perinuclear vesicular structures (Purlyte et al., 2018). These findings are consistent with the idea that pathogenic LRRK2 may cause the phosphorylation of a pool of RAB8A localized to a perinuclear endocytic recycling compartment. Importantly, in both scenarios, this results in the accumulation of phospho-RAB8A in a pericentrosomal/centrosomal area (Figure 11). How such accumulation may cause the observed centrosomal cohesion deficits remains to be determined, but it is tempting to speculate that it involves binding of phospho-RAB8A to RILPL1/RILPL2. These poorly characterized proteins have been shown to selectively bind only to the phosphorylated version of RAB8A (Steger et al., 2017), and have been reported to localize to a pericentrosomal area to modulate ciliogenesis, another centrosome-related event (Schaub and Stearns, 2013).

The centrosomal/pericentrosomal accumulation of phospho-RAB8A in the presence of pathogenic LRRK2 or in the presence of RAB7L1 and wildtype LRRK2 was detected using either sheep polyclonal or rabbit polyclonal antibodies against phospho-RAB8A (Steger et al., 2016; Steger et al., 2017), since at least in our hands, the recently generated monoclonal phospho-RAB8A antibodies (Lis et al., 2018) were unsuitable for immunocytochemistry purposes. The phospho-RAB8A antibodies employed here are known to display cross-reactivity with other RAB proteins which serve as LRRK2 kinase substrates, including RAB3A, RAB8B, RAB10, RAB35, and RAB43 (Steger et al., 2017; Lis et al., 2018). Therefore, it is possible that other phosphorylated RAB proteins may contribute to the centrosomal cohesion phenotype, in particular RAB10, as also shown to interact with RILPL1/RILPL2 only when in its phosphorylated form (Steger et al., 2017). Nevertheless, our data indicate that RAB8A seems to be at least partially responsible, since pericentrosomal/centrosomal RAB8A accumulation was also detected using two distinct knockout-validated antibodies specific against total RAB8A, and since the resulting centrosomal cohesion deficits were at least in part reversed upon knockdown of RAB8A, even though future studies using RAB8A knockout cells will be required to address the full impact of RAB8A on the observed phenotype.

Previous studies have reported that pathogenic LRRK2 disrupts Golgi morphology (MacLeod et al., 2013; Beilina et al., 2014; Purlyte et al., 2018) which may be due to LRRK2-mediated RAB7L1 phosphorylation (Fujimoto et al., 2018). Our data suggest that the centrosomal cohesion deficits mediated by pathogenic LRRK2 are not a downstream effect of altered Golgi morphology, as treatment of control cells with brefeldin A to disrupt Golgi complex integrity was without effect on centrosomal cohesion, even though an intact Golgi is required for the RAB7L1-mediated recruitment and concomitant downstream centrosomal defects mediated by wildtype LRRK2. Similarly, even though LRRK2 phosphorylates RAB7L1 (Fujimoto et al., 2018; Liu et al., 2018), the observed centrosomal cohesion deficits with pathogenic LRRK2 are not due to aberrant RAB7L1 phosphorylation, as also observed upon siRNA of RAB7L1. Rather, they correlate with the abnormal phosphorylation and centrosomal/pericentrosomal accumulation of RAB8A.

Pathogenic LRRK2 has been implicated in causing dysfunctions of various intracellular membrane trafficking steps (Roosen and Cookson, 2016; Madero-Perez et al., 2017). LRRK2-mediated deficits related to lysosomal pathology can be rescued upon RAB7L1 overexpression and are mimicked by RAB7L1 knockdown or knockout, respectively (MacLeod et al., 2013; Wang et al., 2014; Kuwahara et al., 2016). Whilst further work will be required to understand the precise role of RAB7L1 in regulating the LRRK2-linked cellular deficits, it is tempting to speculate that it may play a dual role, with increased levels causing LRRK2 Golgi recruitment, aberrant RAB8A phosphorylation and concomitant centrosomal defects, whilst at the same time also displaying beneficial effects in rescuing select vesicular trafficking deficits induced by pathogenic LRRK2 by currently unknown mechanisms.

The LRRK2-mediated phosphorylation of RAB8A may differentially affect vesicular trafficking events as compared to centrosomal functioning. Our data suggest that phosphorylated RAB8A causes centrosomal deficits in a toxic gain-of-function manner, as knockdown of RAB8A reduces the centrosomal cohesion deficits caused by both pathogenic LRRK2 and RAB7L1-recruited wildtype LRRK2. At the same time, several *in vitro* studies indicate that phosphomimetic RAB8A mutants display decreased interactions with various regulatory proteins expected to lead to RAB8A inactivation (Steger et al., 2016; Liu et al., 2018; Madero-Perez et al., 2018), suggesting that RAB8A phosphorylation may cause a loss-of-function phenotype, possibly in the context of vesicular trafficking events. Both scenarios are conceivable within the same cell, for example via the aberrant localization of phospho-RAB8A in a centrosomal/pericentrosomal location causing centrosomal cohesion deficits, with a concomitant decrease in the amount of membrane-bound RAB8A able to regulate vesicular trafficking events. However, caution is advised with the interpretation of data using phosphomimetic RAB mutants, as such mutants often do not mimick the phosphorylated state of a protein. Indeed, unique protein interactors of the phosphorylated form of RAB8A have only been found when using the phosphorylated protein, but not when using the phosphomimetic versions (Steger et al., 2017). Such inability of phosphomimetic mutants to mimick the phosphorylated state of the protein may also explain why overexpression of phosphomimetic RAB8A mutants does not cause neurotoxicity in primary neurons nor degeneration of dopaminergic neurons *in vivo* (Jeong et al., 2018).

Altogether, our present data indicate that the Golgi recruitment of wildtype LRRK2 by RAB7L1 causes centrosomal alterations by phosphorylation of RAB8A, allowing for the aberrant accumulation of phospho-RAB8A in a pericentrosomal/centrosomal area, and resulting in centrosomal cohesion deficits similar to those caused by pathogenic LRRK2, indicating that such centrosomal cohesion defects may be a common phenotype for a broader spectrum of PD.

## Author Contributions

JM-P, BF, ALO, and EF performed the experiments. EL and VB provided essential cellular reagents. JM-P, BF, ALO, and EF analyzed the data. SH conceived the study, designed the experiments, and analyzed the data. JM-P and SH wrote the paper with contribution from all authors. All authors read and approved the final manuscript.

## Conflict of Interest Statement

The authors declare that the research was conducted in the absence of any commercial or financial relationships that could be construed as a potential conflict of interest. The reviewer PL declared a past co-authorship with several of the authors EL and VB to the handling Editor.
