# Supplementary material for: RAB7L1-Mediated Relocalization of LRRK2 to the Golgi Complex Causes Centrosomal Deficits via RAB8A
Source: Front Mol Neurosci. 2018 Nov 13;11:417. doi: 10.3389/fnmol.2018.00417 (PMC6243087; doi:10.3389/fnmol.2018.00417)
Supplement: Supplementary file 1 [file Data_Sheet_1.PDF]

Appendix

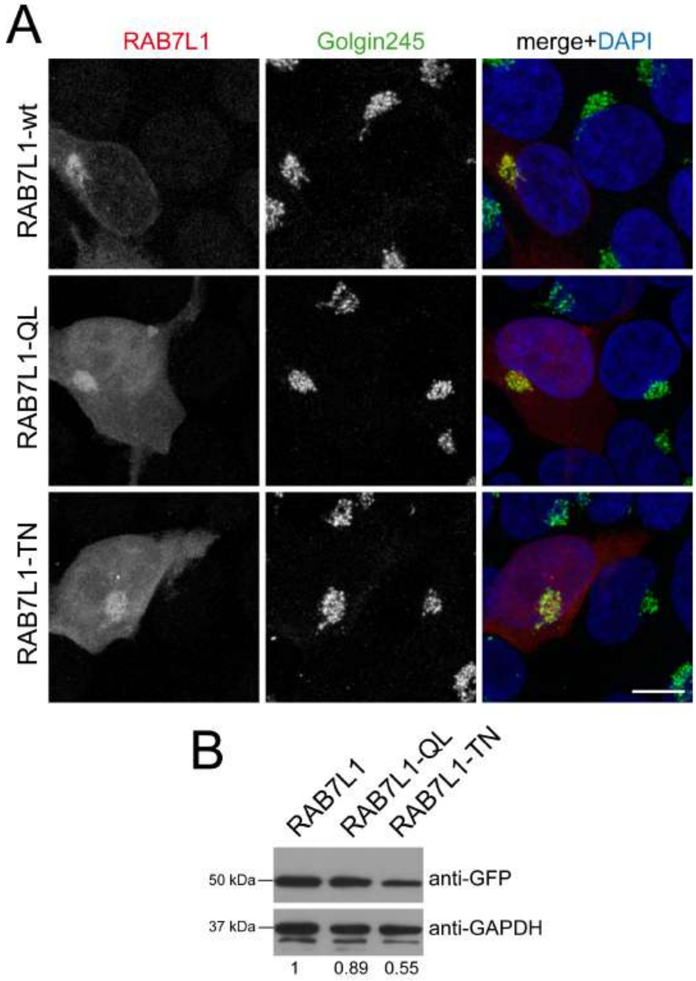

Figure A1. mRFP-tagged wildtype RAB7L1, but not inactive RAB7L1 variants, are localized to the Golgi apparatus. (A) Example of HEK293T cells transfected with mRFP-tagged wildtype or mutant RAB7L1 constructs (red) as indicated, and stained with a trans-Golgi marker antibody (Golgin245, Alexa 488-conjugated secondary antibody, green) and DAPI (blue). Scale bar, 10 µm. (B) Cells were transfected with the indicated GFP-tagged RAB7L1 constructs, and extracts blotted with an anti-GFP antibody or anti-GAPDH as loading control to analyze expression levels of the distinct RAB7L1 variants. Values were normalized to GAPDH, and then normalized to wildtype RAB7L1.

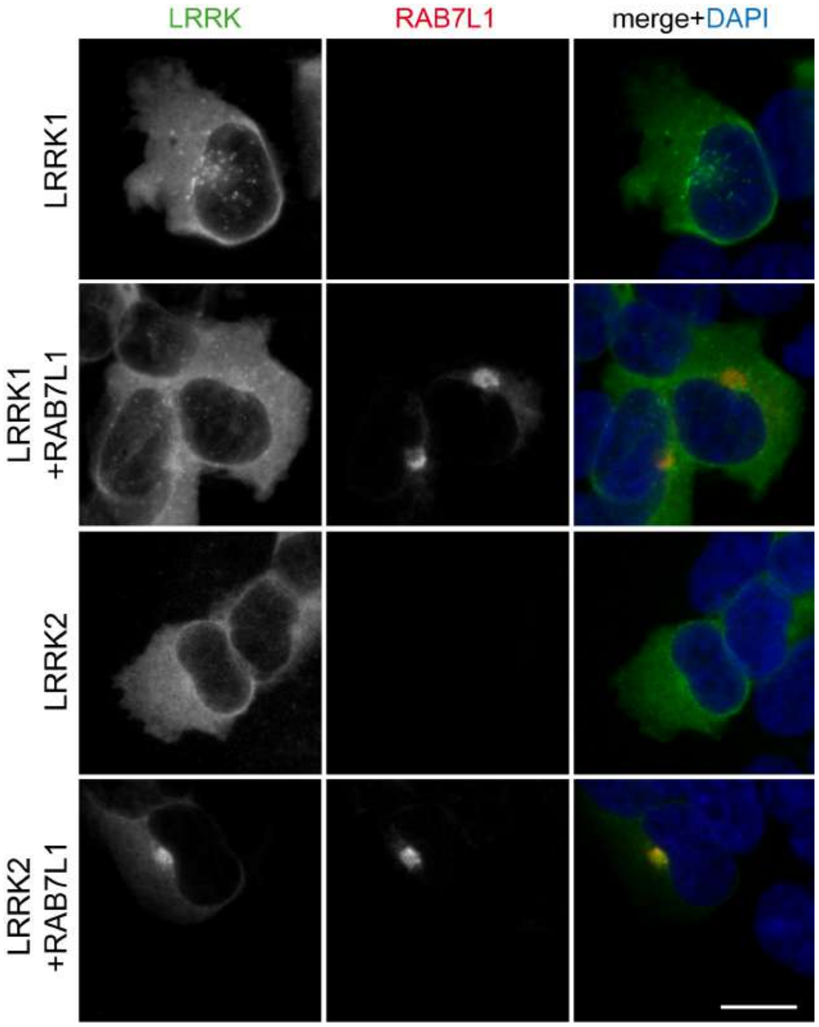

Figure A2. RAB7L1-mediated recruitment of LRRK2, but not of LRRK1. Example of HEK293T cells transfected with either myc-tagged LRRK1 (green), myc-tagged LRRK2, or co-transfected with mRFP-tagged RAB7L1 (red) as indicated, and stained with DAPI (blue). Scale bar, 10 µm.

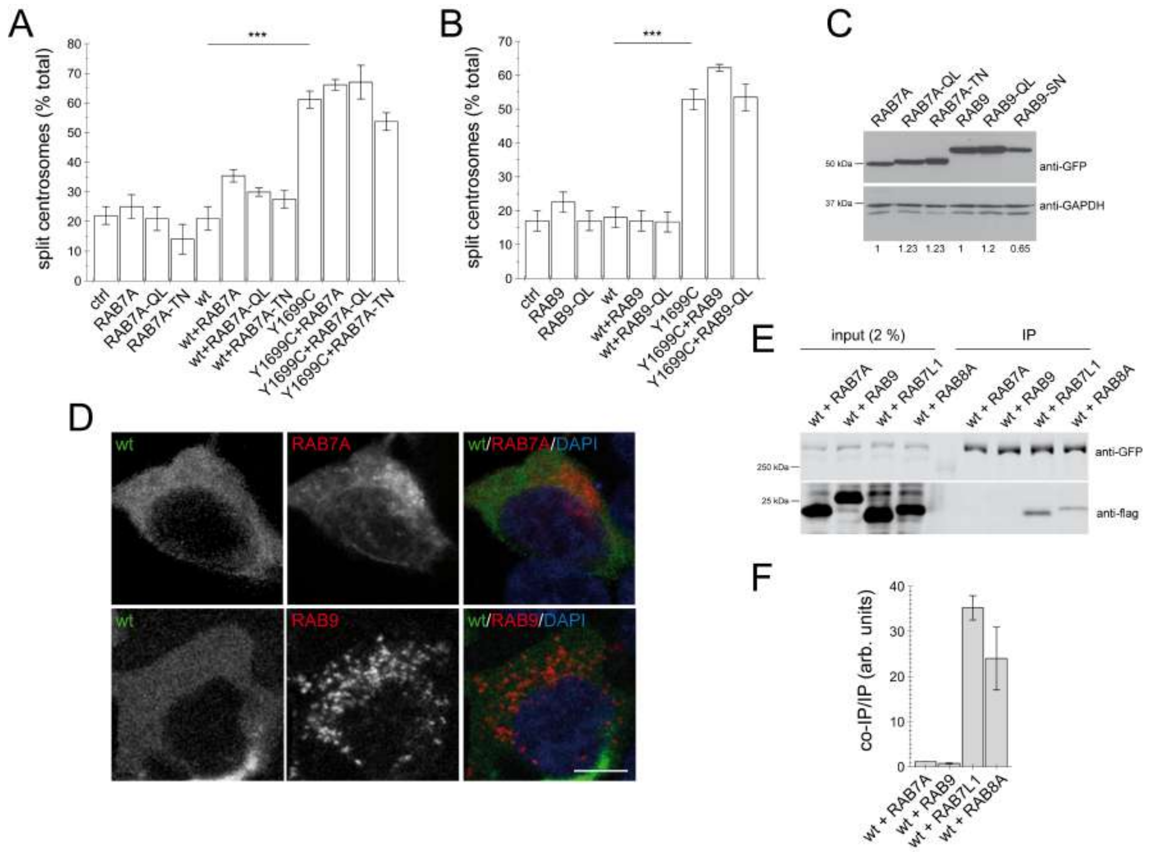

Figure A3. No effect of RAB7A or RAB9 on LRRK2 localization or on LRRK2-mediated centrosomal cohesion deficits. (A) Quantification of the split centrosome phenotype in cells expressing mRFP-tagged RAB7A or mutants thereof, in the absence or presence of wildtype or pathogenic Y1699C-mutant GFP-tagged LRRK2 as indicated. At least 50 transfected cells were analyzed per condition per experiment. Bars represent mean ± s.e.m. (n=3 experiments); ***, $p < 0.005$. (B) Quantification of the split centrosome phenotype in cells expressing ds-Red-tagged RAB9 or mutants thereof, in the absence or presence of wildtype or pathogenic Y1699C-mutant LRRK2 as indicated. Bars represent mean ± s.e.m. (n=3 experiments); ***, $p < 0.005$. At least 50 transfected cells were analyzed per condition per experiment. (C) Cells were transfected with the indicated GFP-tagged RAB7A or RAB9 constructs, and extracts blotted with

an anti-GFP antibody or anti-GAPDH as loading control to analyze expression levels of the distinct RAB variants. Values were normalized to GAPDH, and then normalized to wildtype RAB7A or RAB9, respectively. (D) Example of HEK293T cells expressing GFP-tagged wildtype LRRK2 (green) and either mRFP-RAB7A (red) or dsRed-RAB9 (red), and stained with DAPI (blue). Images are maximal intensity projections of 4 consecutive z-stack images. Scale bar, 5 μm. (E) Cells were cotransfected with wildtype GFP-tagged LRRK2 (wt) and the indicated flag-tagged RAB constructs, and binding between LRRK2 and RABs assessed by co-immunoprecipitation of GFP-tagged LRRK2 with flag-tagged RAB proteins using a polyclonal GFP antibody. Left panel shows inputs, and right panel shows samples after immunoprecipitation, probed for GFP (using a monoclonal GFP antibody) as well as for flag. (F) Quantification of experiments as depicted in (E) were performed by comparing the amount of coimmunoprecipitated RAB proteins to the amount of wildtype LRRK2 in the immunoprecipitation (IP). Data are mean ± s.e.m. (n=3 independent experiments).

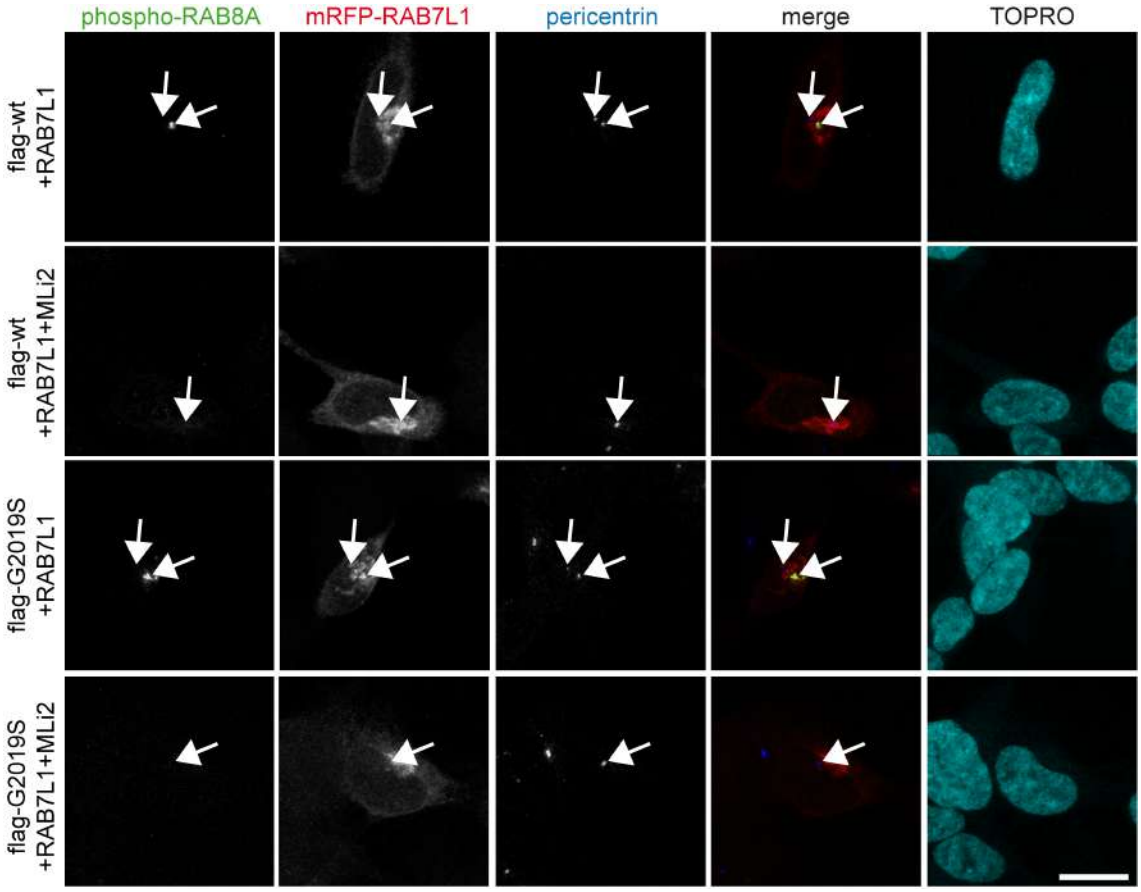

Figure A4. Accumulation of phospho-RAB8A in SH-SY5Y cells expressing RAB7L1. Cells stably expressing flag-tagged wildtype or G2019S LRRK2 were transfected with mRFP-RAB7L1 (red), treated or not treated with 100 nM MLi2 for 60 min prior to immunocytochemistry as indicated, and stained for phospho-RAB8A (Alexa 488-conjugated secondary antibody, green), pericentrin (Alexa 405-conjugated secondary antibody, blue), and TO-PRO-3 (far red fluorescence similar to Alexa 647, pseudo-colored in cyan). Scale bar, 10 μm.

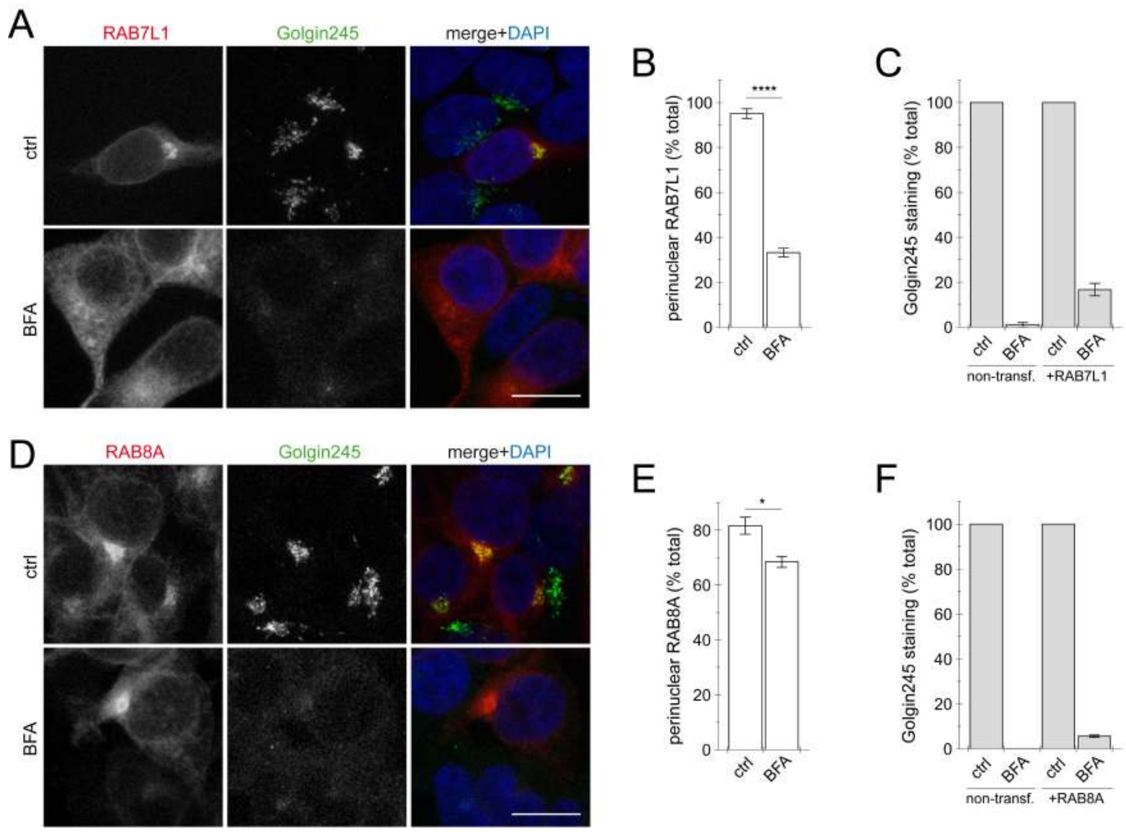

Figure A5. Brefeldin A disrupts the Golgi complex and relocalizes RAB7L1 to the cytosol. (A) Example of HEK293T cells transfected with mRFP-tagged RAB7L1 (red), either non-treated (ctrl) or treated with brefeldin A as indicated, and stained with a trans-Golgi marker antibody (Golgin245, Alexa 488-conjugated secondary antibody, green) and DAPI (blue). Scale bar, 10 μm. (B) Quantification of the percentage of cells with detectable perinuclear RAB7L1 accumulation, in either the absence (ctrl) or presence of brefeldin A as indicated. At least 50 transfected cells were analyzed per condition per experiment. Bars represent mean ± s.e.m. (n=3 experiments); ****, $p < 0.001$. (C) Quantification of the percentage of non-transfected or RAB7L1-transfected cells displaying Golgin245 staining in either the absence or presence of brefeldin A as indicated. At least 50 non-transfected or transfected cells were analyzed per condition

per experiment. Bars represent mean ± s.e.m. (n=3 experiments). (D) Example of HEK293T cells transfected with mRFP-tagged RAB8A (red), either non-treated (ctrl) or treated with brefeldin A as indicated, and stained with a trans-Golgi marker antibody (Golgin245, Alexa 488-conjugated secondary antibody, green) and DAPI (blue). Scale bar, 10 μm. (E) Quantification of the percentage of cells with detectable perinuclear RAB8A accumulation, in either the absence (ctrl) or presence of brefeldin A as indicated. At least 50 transfected cells were analyzed per condition per experiment. Bars represent mean ± s.e.m. (n=3 experiments); *, $p < 0.05$. (F) Quantification of the percentage of non-transfected or RAB8A-transfected cells displaying Golgin245 staining in either the absence or presence of brefeldin A as indicated. At least 50 non-transfected or transfected cells were analyzed per condition per experiment. Bars represent mean ± s.e.m. (n=3 experiments).

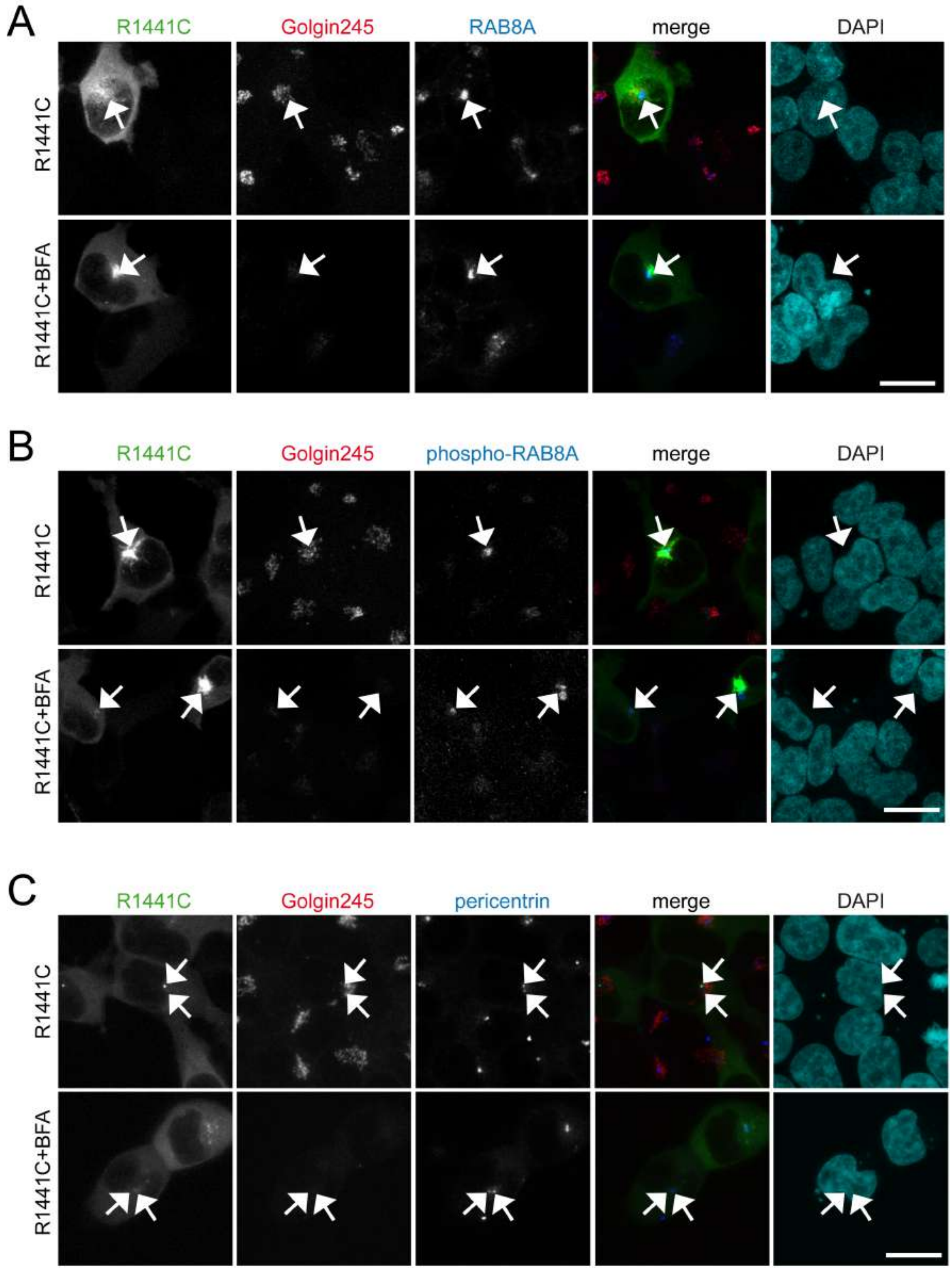

Figure A6. Integrity of the Golgi complex is not required for the pathogenic R1441C LRRK2-mediated phospho-RAB8A accumulation and centrosomal cohesion deficits. (A) HEK293T cells were transfected with R1441C-mutant LRRK2 (green), either

treated or non-treated with brefeldin A (BFA; 7.5 μg/ml) for 3 h, and stained with a knockout-validated rabbit monoclonal anti-RAB8A antibody (Alexa 647-conjugated secondary antibody, pseudo-colored in blue), a trans-Golgi marker antibody (Golgin245, Alexa 594-conjugated secondary antibody, red) and DAPI (pseudo-colored in cyan). Scale bar, 10 μm. (B) Cells were transfected with R1441C-mutant LRRK2 (green), and either treated or non-treated with brefeldin A as described above before staining with a rabbit polyclonal phospho-RAB8A antibody (Alexa 647-conjugated secondary antibody, pseudo-colored in blue), a trans-Golgi marker antibody (Golgin245, Alexa 594-conjugated secondary antibody, red) and DAPI (pseudo-colored in cyan). Scale bar, 10 μm. (C) Cells were transfected with R1441C-mutant LRRK2 (green), and either treated or non-treated with brefeldin A as described above before staining with a pericentrin antibody (Alexa 647-conjugated secondary antibody, pseudo-colored in blue), a trans-Golgi marker antibody (Golgin245, Alexa 594-conjugated secondary antibody, red) and DAPI (pseudo-colored in cyan). Scale bar, 10 μm.

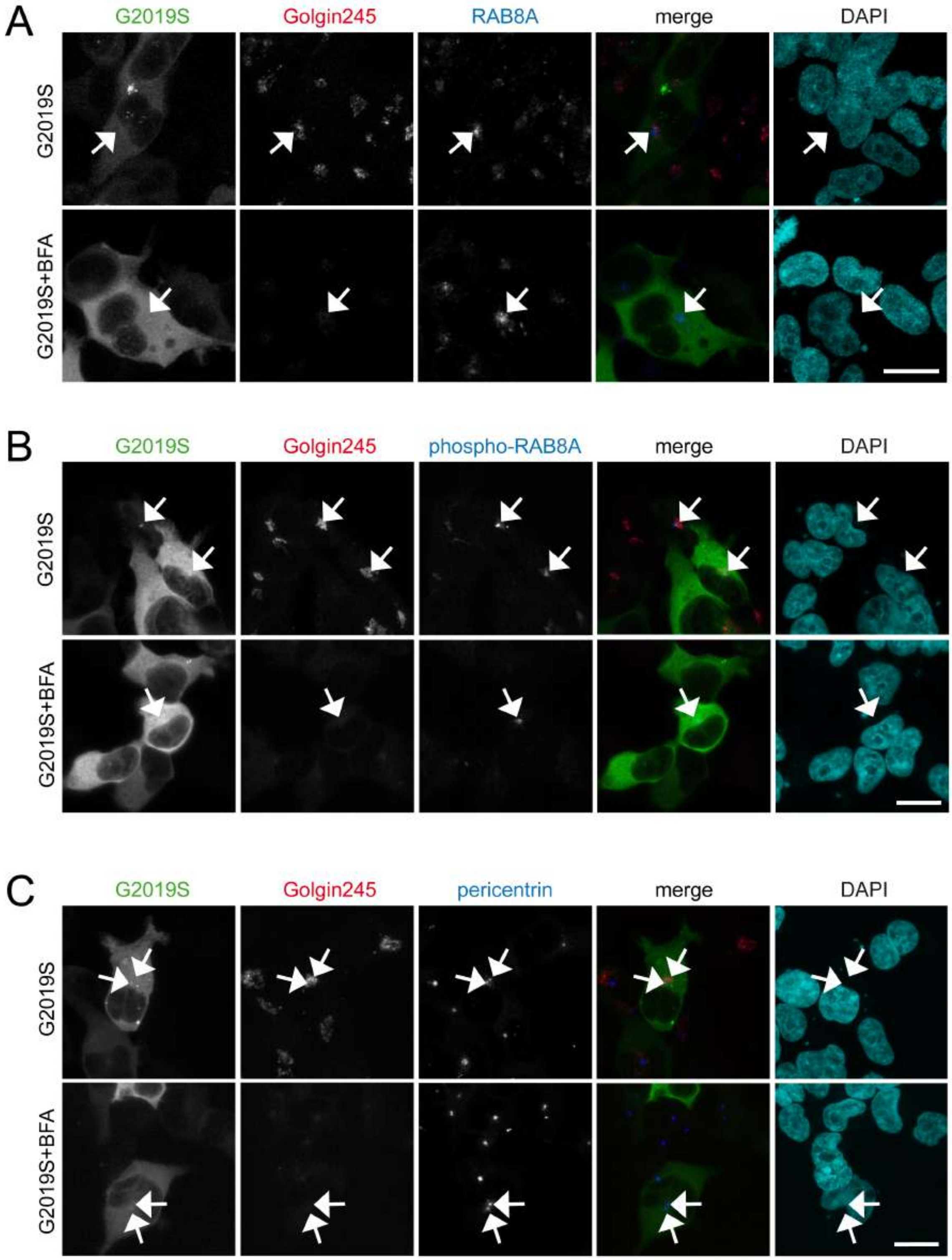

Figure A7. Integrity of the Golgi complex is not required for the pathogenic G2019S LRRK2-mediated phospho-RAB8A accumulation and centrosomal cohesion deficits. (A) HEK293T cells were transfected with G2019S-mutant LRRK2 (green), either

treated or non-treated with brefeldin A (BFA; 7.5 µg/ml) for 3 h, and stained with a knockout-validated rabbit monoclonal anti-RAB8A antibody (Alexa 647-conjugated secondary antibody, pseudo-colored in blue), a trans-Golgi marker antibody (Golgin245, Alexa 594-conjugated secondary antibody, red) and DAPI (pseudo-colored in cyan). Scale bar, 10 µm. (B) Cells were transfected with G2019S-mutant LRRK2 (green), and either treated or non-treated with brefeldin A as described above before staining with a rabbit polyclonal phospho-RAB8A antibody (Alexa 647-conjugated secondary antibody, pseudo-colored in blue), a trans-Golgi marker antibody (Golgin245, Alexa 594-conjugated secondary antibody, red) and DAPI (pseudo-colored in cyan). Scale bar, 10 µm. (C) Cells were transfected with G2019-mutant LRRK2 (green), and either treated or non-treated with brefeldin A as described above before staining with a pericentrin antibody (Alexa 647-conjugated secondary antibody, pseudo-colored in blue), a trans-Golgi marker antibody (Golgin245, Alexa 594-conjugated secondary antibody, red) and DAPI (pseudo-colored in cyan). Scale bar, 10 µm.

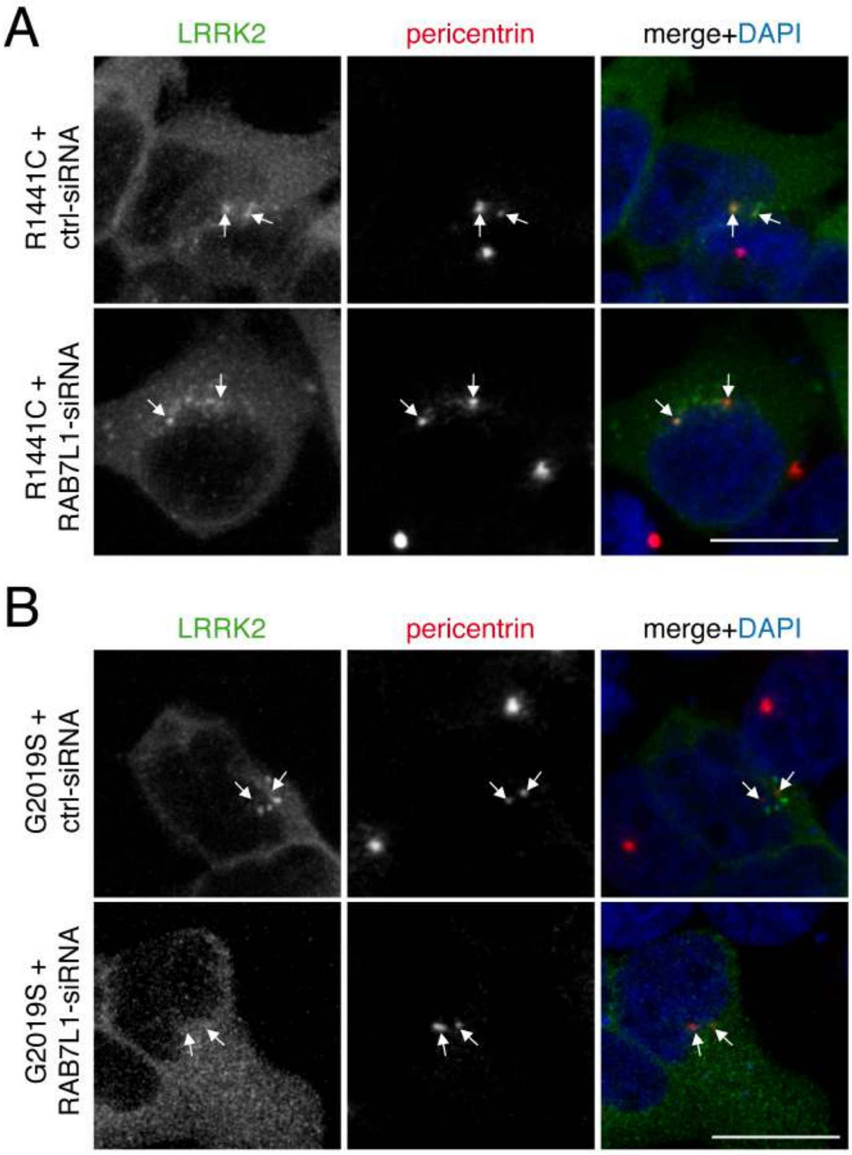

Figure A8. Knockdown of RAB7L1 does not alter the centrosomal cohesion deficits mediated by R1441C or G2019S LRRK2. (A) Example of HEK293T cells co-transfected with GFP-tagged R1441C mutant LRRK2 (green) and either ctrl-siRNA or RAB7L1-siRNA as indicated, and stained with pericentrin antibody (Alexa 647-conjugated secondary antibody, pseudo-colored in red) and DAPI (blue). Scale bar, 10 μm. (B) Example of HEK293T cells co-transfected with GFP-tagged G2019S mutant LRRK2 (green) and either ctrl-siRNA or RAB7L1-siRNA as indicated, and stained with

pericentrin antibody (Alexa 647-conjugated secondary antibody, pseudo-colored in red) and DAPI (blue). Scale bar, 10 μm.
